# Modeling Friedreich’s ataxia with Bergmann glia-enriched human cerebellar organoids

**DOI:** 10.1038/s42003-026-10180-5

**Published:** 2026-05-08

**Authors:** Seungmi Ryu, Jason Inman, Hyenjong Hong, Vukasin M. Jovanovic, Qiang Chen, Yeliz Gedik, Yogita Jethmalani, Inae Hur, Majid Harouni, Ty Voss, Justin Lack, Jack Collins, Pinar Ormanoglu, Anton Simeonov, Carlos A. Tristan, Ilyas Singeç

**Affiliations:** 1 National Center for Advancing Translational Sciences (NCATS), Stem Cell Translation Laboratory (SCTL), National Institutes of Health (NIH), Rockville, MD USA; 2 National Institute of Allergy and Infectious Diseases (NIAID), Collaborative Bioinformatics Resource (NCBR), National Institutes of Health (NIH), Bethesda, MD USA

**Keywords:** Stem-cell differentiation, Disease model, Developmental neurogenesis, Neural stem cells

## Abstract

The human cerebellum is implicated in various neurological and psychiatric diseases, but its complex development and cellular diversity have posed challenges for in vitro modeling. Here, we report the generation of human induced pluripotent stem cell (iPSC)-derived cerebellar organoids (hCBOs) that are characterized by induction of rhombomere 1 (R1) cellular identity and followed by derivation of typical neuronal and glial cell types of the cerebellum. In contrast to forebrain organoids with multiple neural rosettes and inside-out neuronal migration, hCBOs develop a germinal zone on the outermost surface of the organoids with outside-in neuronal migration. These hCBOs produce various neuronal cell types resembling granule neurons, Purkinje cells, Golgi neurons, and deep cerebellar nuclei. By using a glial induction strategy, we generate Bergmann glial cells (BGCs) that serve as scaffolds for migratory granule cells and enhance electrophysiological activity of the hCBOs. Furthermore, by generating hCBOs from patients with Friedreich’s ataxia (FRDA), we reveal disease-specific phenotypes that can be reversed by histone deacetylase (HDAC) inhibitors and gene editing by CRISPR-Cas9. Taken together, our advanced hCBO model provides new opportunities to investigate the mechanisms of cerebellar ontogenesis and utilize patient-derived iPSCs for translational research.

## Abstract (web summary)

Bergmann-glia-enriched human cerebellar organoids model Friedreich’s ataxia, recapitulating cerebellar histogenesis and disease phenotypes reversible by HDAC inhibition and CRISPR-based gene correction.

## Introduction

The mammalian cerebellum is a unique brain region that plays important roles not only in motor coordination, balance, and posture but also in cognitive function (*i.e*., learning and memory) and processing of language and emotions^1^. The highly complex anatomy and physiology of the human cerebellum can be affected by a broad range of early- and late-onset diseases with neurodevelopmental, genetic, and neurodegenerative causes (e.g., FRDA, Joubert syndrome, Dandy-Walker malformation, drug intoxication)^2^. In addition, the cerebellum is affected in neuropsychiatric disorders, including autism spectrum disorders, schizophrenia, and depression^3^

During the early stages of brain development, the cerebellum originates from R1, a defined region at the midbrain-hindbrain boundary that is caudal to the isthmic organizer^4^. The R1 region gives rise to the cerebellar plate with two distinct neural precursor populations forming the rhombic lip (RL) and the ventricular zone (VZ) of the 4th ventricle, which together will contribute to the diverse cell types of the cerebellum^5^. The RL gives rise to all excitatory cells, including granule cells (GCs), unipolar brush cells, and the glutamatergic deep cerebellar nuclei^5^. The VZ produces Purkinje cells, Golgi cells, basket cells, and the GABAergic deep cerebellar nuclei neurons^5^.

One of the characteristic features of cerebellar development is that dorsally located RL cells migrate tangentially and generate the external germinal layer (EGL), a transient layer of progenitors at the pial surface that produces the vast majority of GCs, which then migrate in an outside-in direction to find their final destination in the internal granular layer (IGL) in the more mature cerebellum^6^. The emergence of BGCs, a specialized unipolar cell type with long processes, is important in guiding migratory GCs from the EGL toward the IGL^7^. BGCs are thought to originate from radial glial progenitors of the cerebellar plate ventricular zone (CPVZ), specifically from the cerebellar plate neuroepithelium (CPNE)^7^. Disruption of cell migration processes in the developing cerebellum has been linked to neurodevelopmental disorders, cerebellar ataxias, and impaired motor function^8,9^.

Human cerebellar development and the many diseases that affect the cerebellum, particularly rare genetic diseases, remain largely understudied. Reliable in vitro systems that faithfully recapitulate the key aspects of human cerebellar development are urgently needed for basic and translational research as substantial genetic, anatomic, and physiological differences exist between humans and animal models^1,10–15^. Here, we developed a new 60-day method for deriving hCBOs from iPSCs exhibiting critical features of cerebellar development. These hCBOs showed distinct formation of a germinal layer on the organoid surface, well-developed BGCs, and typical outside-in migration of GCs. Furthermore, we generated hCBOs from patients with FRDA, the most common inherited ataxia caused by abnormal GAA triplet-repeats in the FXN gene that affects 1 in 50,000 people globally^16,17^. The use of patient-derived iPSC lines with short and long GAA repeats, which differentially impact frataxin expression levels and correlate with disease severity in the clinic, enabled the identification of disease phenotypes that were reversed by chemical and genetic strategies.

## Results

### A cerebellar organoid model with outside-in migration

**Fig. 1:**
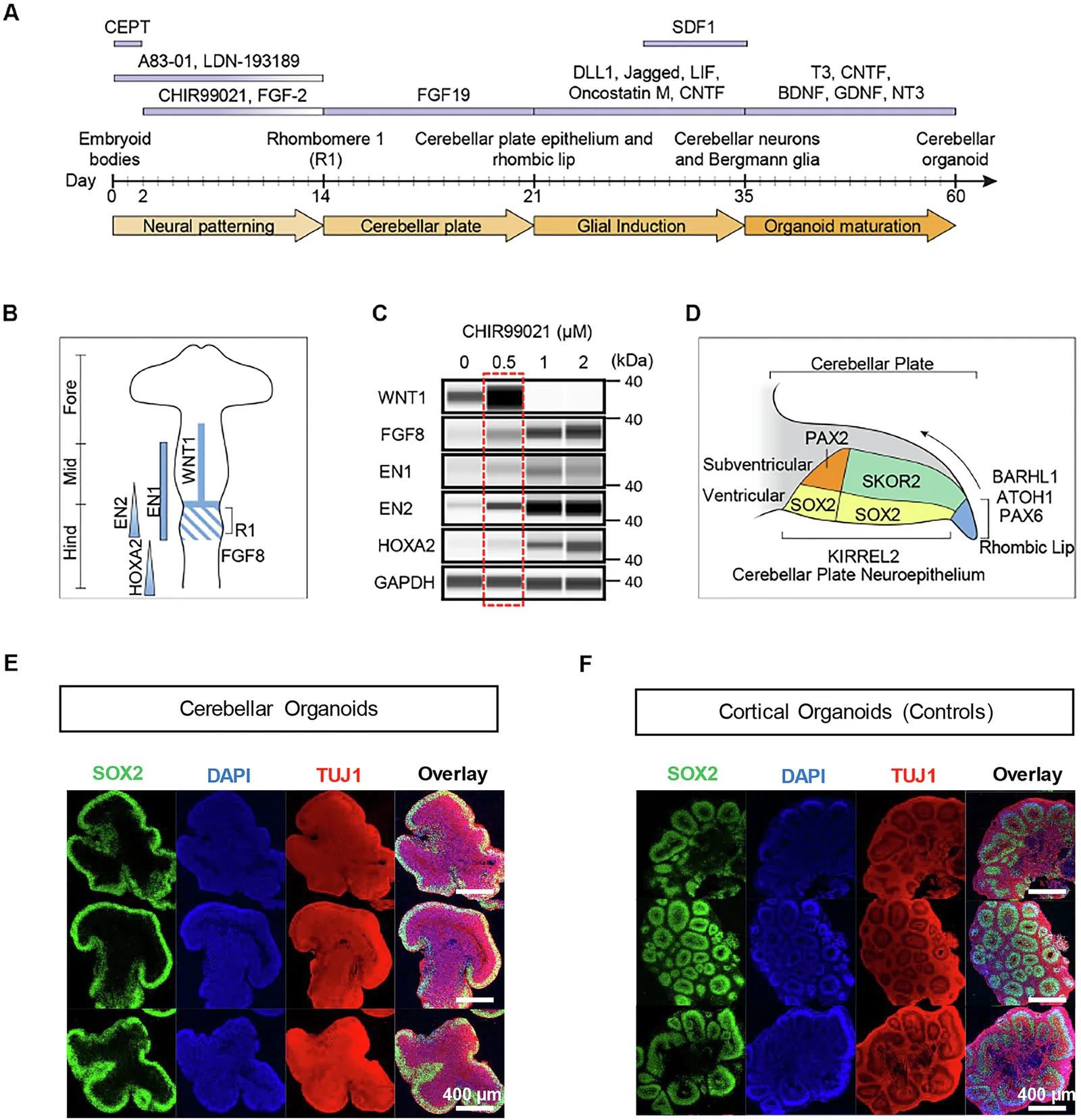
Establishment and characterization of hCBOs. **A** Overview of the stepwise protocol for cerebellar organoid formation. **B** Gene expression pattern during regional specification of the embryonic brain. The cerebellum arises from the R1 region caudally adjacent to the mid-hindbrain boundary. **C** R1 patterning optimization via WNT activator CHIR99021 dose-response experiments in day 14 organoids. The red box indicates the optimal concentration for generating the R1 signature. **D** Schematic diagram showing the ventricular zone (VZ) and subventricular zone (SVZ) of cerebellar primordia, including the cerebellar plate neuroepithelium (CPNE) and the rhombic lip (RL). The schematic diagram was adapted from previous studies^10,25^. **E** Overview of three representative examples of iPSC-derived hCBOs (GM25256, day 21). Note the distinct formation of VZ (SOX2-expressing cells) on the organoid surface. Neuronal cells were immunostained using an antibody against TUJ1. **F** Representative images of three iPSC-derived cortical organoids (GM25256, day 35) with multiple SOX2^+^ expressing neural rosettes inside the organoids.

Considering the unique features of cerebellar development and guided by the principles of early patterning along the neuraxis (dorso-ventral and anterior-posterior), we developed a stepwise 60-day differentiation protocol that consists of four stages characterized by neural induction and R1 specification, formation of the cerebellar plate, emergence of BGCs, and organoid maturation (Fig. 1A). First, human embryonic stem cell (ESC; WA09) and different iPSC lines (GM25256, GM23404, GM23903, NCRM5) were dissociated, and a defined number of cells were aggregated to form embryoid bodies (EBs) in chemically defined medium supplemented with the CEPT small molecule cocktail (Fig. 1A)^18^. We previously demonstrated that the CEPT cocktail is essential for stress-free passaging of human pluripotent stem cells (hPSCs) and for promoting optimal aggregation of hPSCs into EBs^19^. The first stage of the protocol (day 0–14) was aimed at neural induction and patterning into R1, which gives rise to the entire cerebellum during fetal brain development. Of note, R1 is the most anterior segment of the hindbrain and the only hindbrain region that does not express HOX genes^20^. Activation of the WNT pathway has a caudalizing effect during early neural patterning^21^ and we performed dose-response experiments using the well-established WNT pathway activator CHIR99021. This treatment induced the expression of WNT1 and FGF8 (Fig. 1B, C), which are critical for development of R1^22,23^. These experiments also revealed that the administration of 0.5 µM CHIR99021 was optimal to induce R1 identity, as demonstrated by the expression of transcription factors EN1 and EN2 (Fig. 1C). Importantly, HOXA2, the most rostrally expressed HOX gene^24^, was not induced at 0.5 µM CHIR99021 (Fig. 1C), whereas higher concentrations of CHIR99021 (1 µM and 2 µM) enhanced caudalization as indicated by suppressed WNT1 and increased HOXA2 expression. Omitting CHIR99021 from the cultures showed lack of caudalization (Fig. 1C).

After inducing R1-like cellular identity, in the second stage of our protocol (day 14–21) cells were specified to generate the cerebellar plate by exposure to FGF19 (Fig. 1A). The cerebellar plate contains the two primordial regions of the early developing cerebellum, the cerebellar plate neuroepithelium (CPNE) and the RL. The cells of these subregions express distinct markers including PAX2, SKOR2, KIRREL2 for subventricular zone (SVZ) cells originating from the CPVZ^10^. In contrast, cells expressing PAX6, BARHL1, ATOH1 represent cell types of the RL (Fig. 1D)^10,25,26^.

To directly compare forebrain organoids to hCBOs, in control experiments, we generated cortical organoids following an established protocol^27^. Immunocytochemical analysis of hCBOs at day 21 showed cerebellar organization with SOX2^+^ precursors located exclusively on the organoid surface and TUJ1^+^ neuronal cells occupying the inner region (Fig. 1E). In contrast to hCBOs, cortical organoids generated SOX2^+^ precursor cells in the inner region and TUJ1^+^ neurons on the outer region of neural rosettes (Fig. 1F). Hence, this direct comparison demonstrated that our protocol generates hCBOs with a distinct anatomical architecture and are different from cortical organoids.

To investigate neuronal cell migration patterns in hCBOs, we performed pulse-chase birth dating experiments using 5-bromo-2′-deoxyuridine (BrdU), a thymidine analog that is incorporated into the DNA of dividing cells and can be detected by immunohistochemistry. hCBOs were briefly exposed to BrdU for 2 h at either day 28 or 32 to label proliferative cells on the organoid surface, followed by immunohistochemical analysis at day 35 (Fig. S1A). Quantitative analysis and replicate experiments confirmed that early-born BrdU-labeled TUJ1^+^ neuronal cells (exposed to BrdU at day 28) migrated more extensively (relative to the organoid surface) than those labeled at the later time point (day 32) (Fig. S1B, S1C). These findings support the notion that time-dependent outside-in neuronal migration occurs in our hCBOs.

### Generation of Bergmann glia and the diversity of neuronal cell types of the cerebellum

**Fig. 2:**
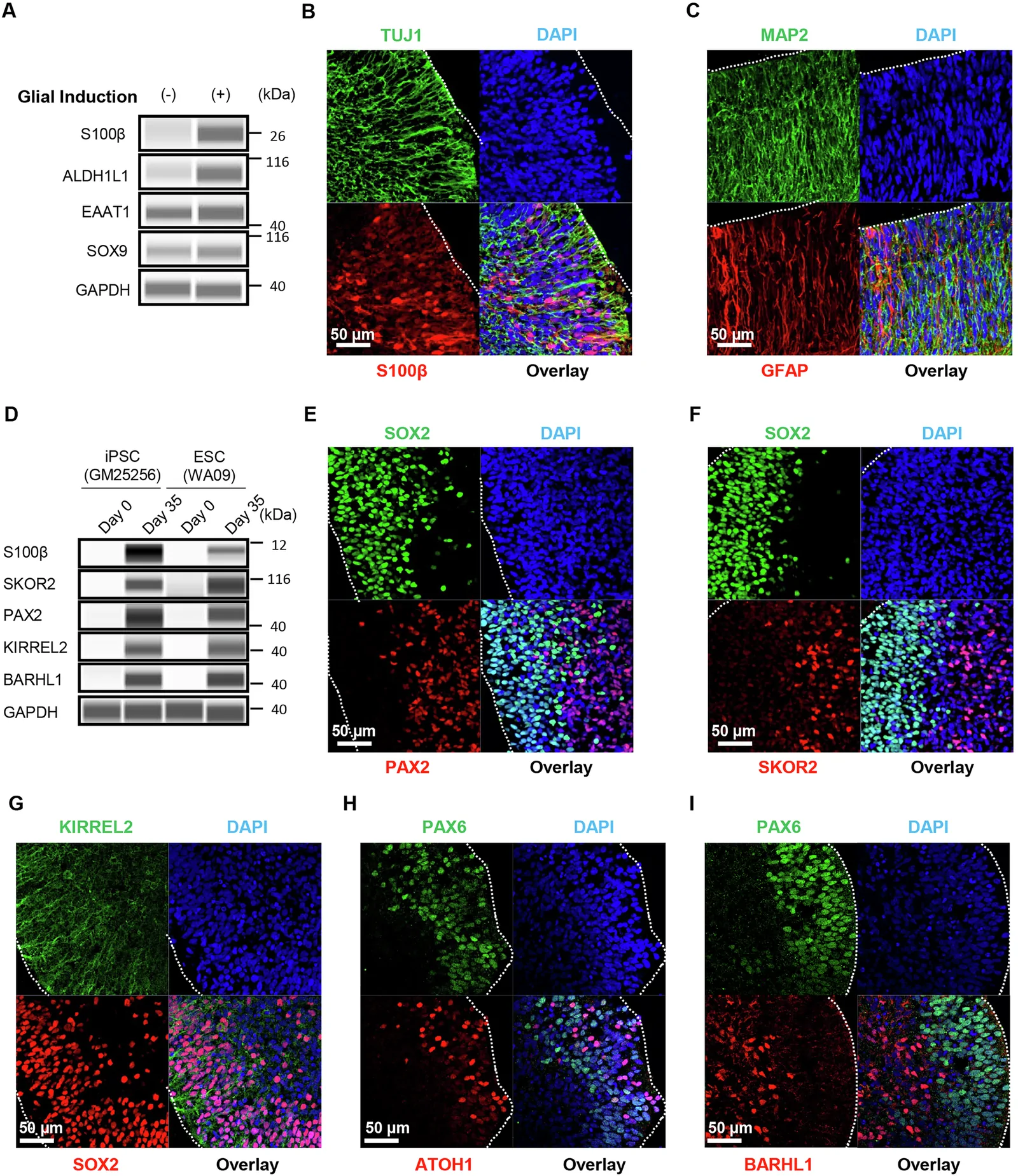
Generation and characterization of Bergmann glia and other cerebellar progenitors. **A** Western blot analysis showing the effects of glial induction on upregulation of glial markers. **B**, **C** Glial induction results in prominent expression of S100β at day 35 and GFAP at day 60 hCBOs. Note the long radial processes of BGCs that are immunopositive for GFAP. **D** Western blot analysis showing expression of typical markers expressed by cells originating from the CPNE (S100β, SKOR2, PAX2, KIRREL2) or RL (BARHL1). **E**, **F** Immunostaining showing formation of a SOX2^+^ germinal zone and CPVZ derivatives in deeper layers expressing PAX2^+^ and SKOR2^+^. **G** Early Purkinje cell marker KIRREL2 stains radially-oriented long processes. **H**, **I** Double immunostainings showing distinct RL-like cells expressing PAX6, ATOH1, and BARHL1. These findings suggest that PAX6-expressing cells represent an earlier developmental cell state compared to cells expressing ATOH1 or BARHL1. White dashed lines in (**B**, **C**), (**E**, **F**), and (**G**–**I**) indicate the surface of hCBOs.

Normal development and the proper formation of the cerebellar cortex depends on BGCs with long radial processes that serve as scaffolds for migratory GCs^6^. Because the spontaneous emergence of glial cells in organoids is a protracted process and can take 3–6 months^27,28^, in the third stage of our protocol (day 21–35) we took advantage of a combination of glial-inducing factors (Fig. 1A) that we previously utilized as part of a different protocol to efficiently generate radial glial and astrocytes in a monolayer model^29^. We also added the chemokine stromal-derived factor 1-alpha (SDF1α), which has important roles in regulating GC migration^30^. This efficiently produced glial cells with long radial processes resembling BGCs and showed expression of typical glial markers by day 35 including S100β, ALDH1L1, EAAT1, SOX9 and eventually abundant expression of GFAP in day 60 hCBOs (Fig. 2A–C, S2).

At day 21, cells expressing CPNE lineage markers (SKOR2, PAX2) were found adjacent to SOX2^+^ precursor cells (Fig. S3A, S3B). EN2 expression indicated the presence of early cerebellar lineage cells in hCBOs at this stage, consistent with a previous study (Fig. S3C)^31^. PAX2 and ATOH1 immunostaining confirmed the emergence of CPNE- and RL-like regions within the hCBOs (Fig. S4). By day 35, adjacent to SOX2^+^ and PAX6^+^ precursors, subpopulations of neuronal progenitors expressing distinct markers were detected by immunohistochemistry and Western blot analysis (Fig. 2D-I, see also schematic in Fig. 1D), including PAX2, SKOR2, KIRREL2 labeling the CPNE SVZ and BARHL1, ATOH1 labeling the RL (Fig. 2E-I). The overall data confirmed the presence of two primordial regions of the early developing cerebellum (CPNE and RL) by day 35 in hCBOs.

**Fig. 3:**
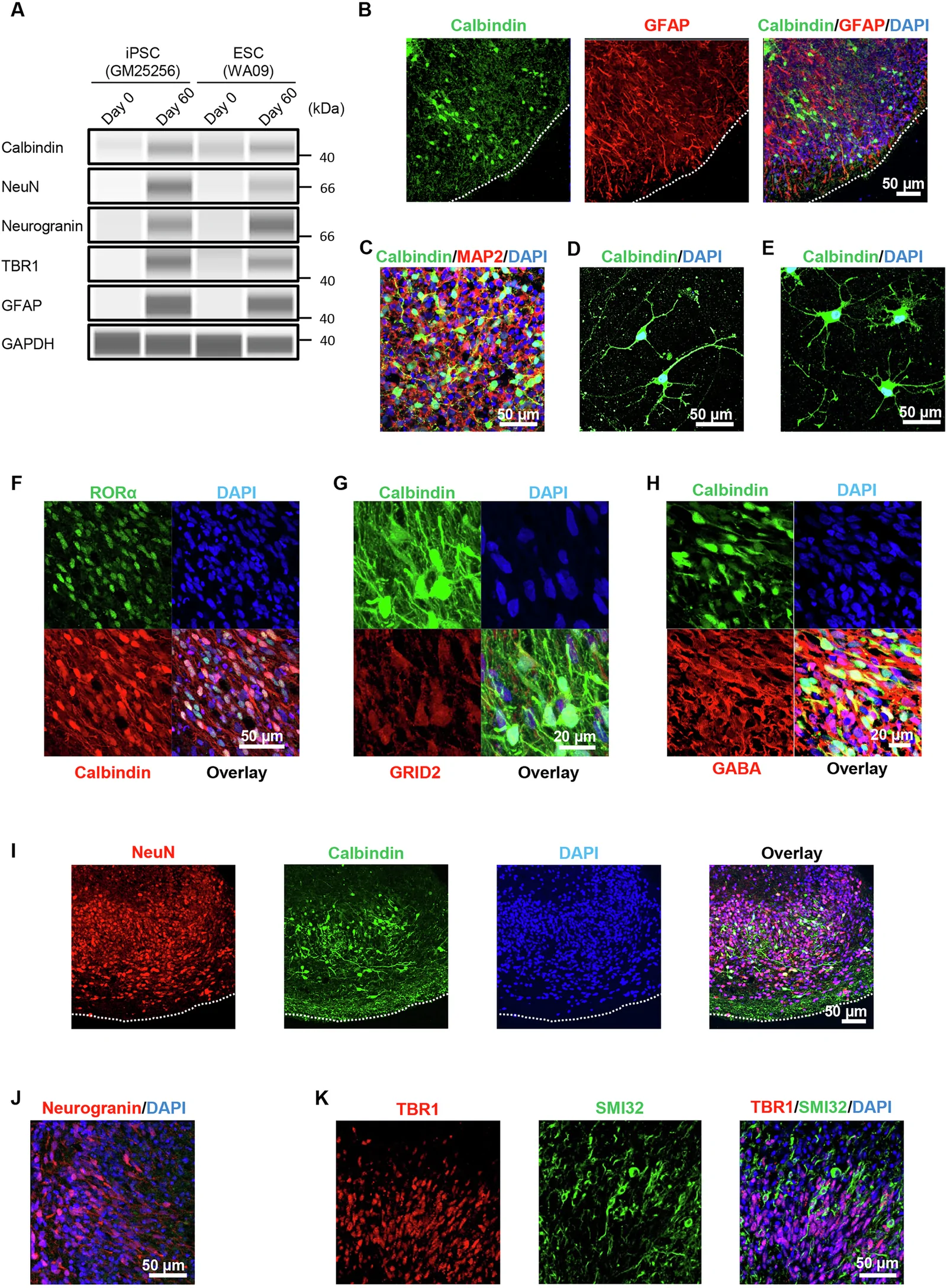
Immunophenotypic characterization of cerebellar neurons (day 60). **A** Western blot analysis demonstrating robust expressions of various cell type-specific cerebellar markers in hCBOs derived from an iPSC line (GM25256) and human ESC line (WA09). **B** Expression of calbindin by Purkinje cells and expression of GFAP by BGCs. **C** Densely packed neuronal cells expressing calbindin and neuronal marker MAP2. **D**, **E** Purkinje cells expressing calbindin observed in 2D tissue culture 30 days after dissociation of day-60 hCBOs. Note the complex neuronal morphologies of early Purkinje cells. **F**–**H** Co-expression of calbindin with RORα, GRID2, and GABA in day 60 hCBOs. **I** Visualization of large numbers of migratory GCs (NeuN^+^) and Purkinje cells (calbindin^+^). **J** Expression of neurogranin by Golgi cells. **K** Immunostaining for TBR1 and SMI32 labeling neurons of deep cerebellar nuclei. White dashed lines in (**B**) and (**I**) indicate the outer layer of hCBOs.

In the final step of our protocol (day 35–60), we matured hCBOs by adding a cocktail of neurotrophic factors (*i.e.*, CNTF, BDNF, GDNF, and NT3) and T3 thyroid hormone (Fig. 1A). By day 60, Western blot and immunohistochemical analyses confirmed the expression of cell type-specific markers (Fig. 3A-K). Purkinje cells are GABAergic neurons and express the calcium-binding protein calbindin^10,13^. We observed immature Purkinje neuron-like cells with various morphologies and immunoreactivity for calbindin, RORα, GRID2, and GABA (Fig. 3B-H). The neuronal protein NeuN is expressed by postmitotic cerebellar GCs but not in Purkinje cells^32^. Accordingly, we found large numbers of NeuN^+^ GCs that were devoid of calbindin expression (Fig. 3I). Neurogranin is expressed by cerebellar Golgi cells^33^ and we found a subset of neurons expressing this marker (Fig. 3J). Neurons of the deep cerebellar nuclei co-express TBR1 and SMI32^10,34^, and cells expressing these markers were detected in hCBOs as well (Fig. 3K). Taken together, these data demonstrate that our 60-day protocol generated hCBOs composed of various types of cerebellar neurons and BGCs.

### Structural and functional consequences of glial induction in hCBOs

**Fig. 4:**
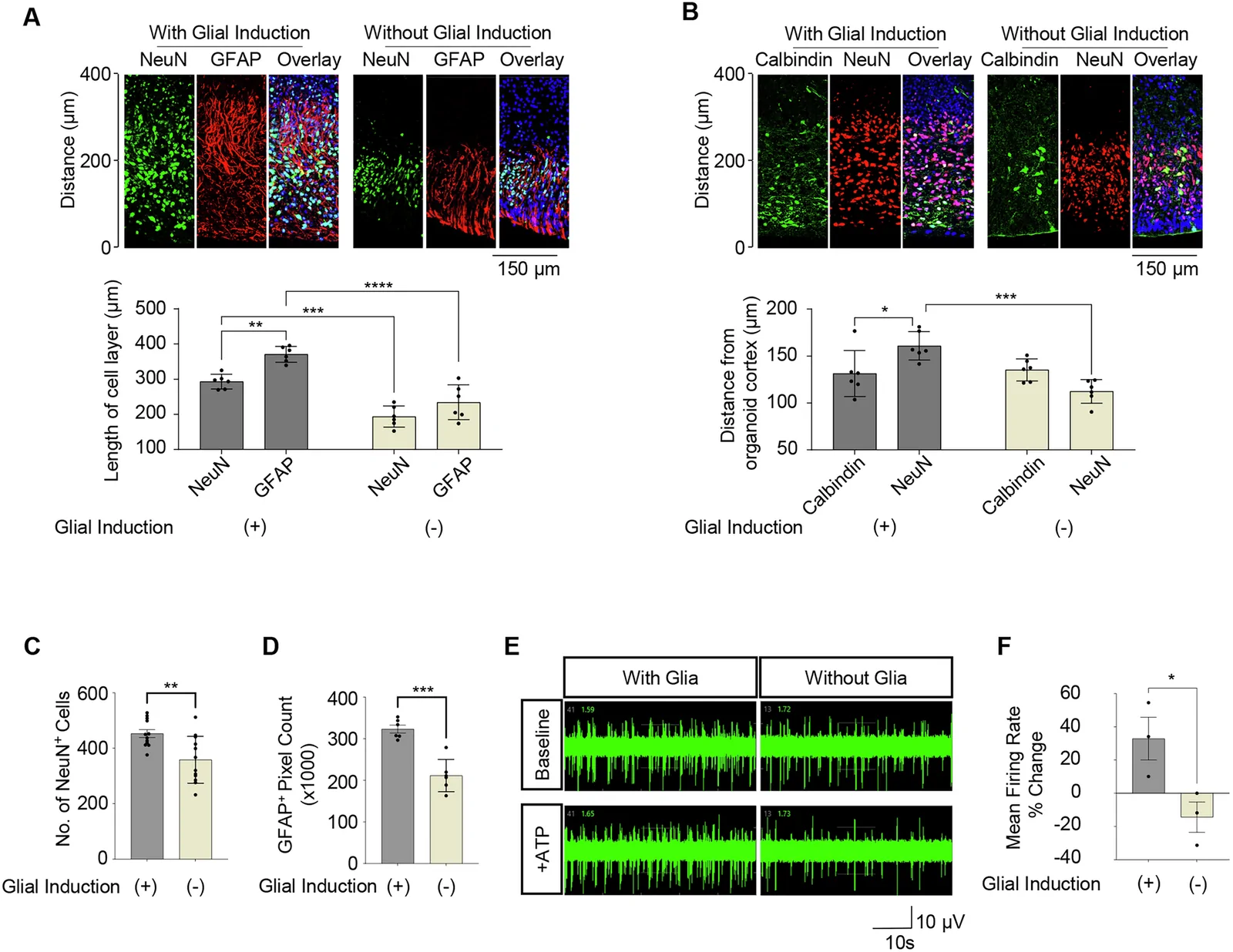
Glial induction and functional effects of BGCs. **A**–**D** Comparison of hCBOs with and without glial induction, quantification of neuronal migration, and quantitative analysis of NeuN and GFAP immunostainings. GCs were visualized using an antibody against NeuN, Purkinje cells were detected by calbindin expression, and BGCs were labeled by GFAP expression. Note the positive effect of glial induction on GFAP expression, layer formation, and more extensive neuronal migration. **E**, **F** MEA analysis of hCBOs (day 80) stimulated with 100 μM ATP. *n* = 6 in **A**, **B**, **D**, *n* = 12 in **C**, and *n* = 3 in **F**. Error bar, mean ± SEM. *p < 0.05. **p < 0.01. ***p < 0.001. ****p < 0.0001.

To characterize the role of BGC-like cells, we developed an image-based machine-learning approach to measure cell migration and localization of GCs, BGCs, and Purkinje neurons (Fig. 4A-D, S5). When hCBOs were generated either with or without glial induction and subjected to quantitative morphological analysis, a marked difference was observed between the two groups. Organoids exposed to glial-inducing factors showed more prominent GFAP^+^ structures including long processes covering larger areas as compared to non-treated hCBOs (Fig. 4A). Accordingly, NeuN^+^ GCs were spread over a larger area suggesting more efficient outside-in migration of GCs guided by BGC scaffolds (Fig. 4A, B). Moreover, quantification of NeuN^+^ GCs and GFAP^+^ BGCs supports the notion of increased numbers of GCs due to glial induction (Fig. 4C, D), which is consistent with the known supporting roles of BGCs for GC survival and proliferation^35,36^.

The presence of BGCs can modulate the electrophysiological activity of cerebellar neurons, including Purkinje cells, upon ATP stimulation^37^. Previous work has shown that ATP stimulation evokes a Ca^2+^ response in BGCs by transiently increasing their K^+^ uptake. This resulting reduction in extracellular K^+^ concentration increases Purkinje cell excitability and increases their spike activity^37^. Microelectrode array (MEA) analysis of day 80 organoids demonstrated that while both organoids, either with or without glial induction, exhibited spontaneous electrical activity, only hCBOs that underwent glial induction exhibited significant changes in firing rate after the addition of ATP (Fig. 4E, F). Overall, these findings demonstrated that the presence of BGC-like cells in hCBOs supported neuronal migration during early layer formation and electrophysiological response to ATP as a biological stimulus.

### Time-course transcriptomic analysis of hCBOs from healthy donors and patients with FRDA

**Fig. 5:**
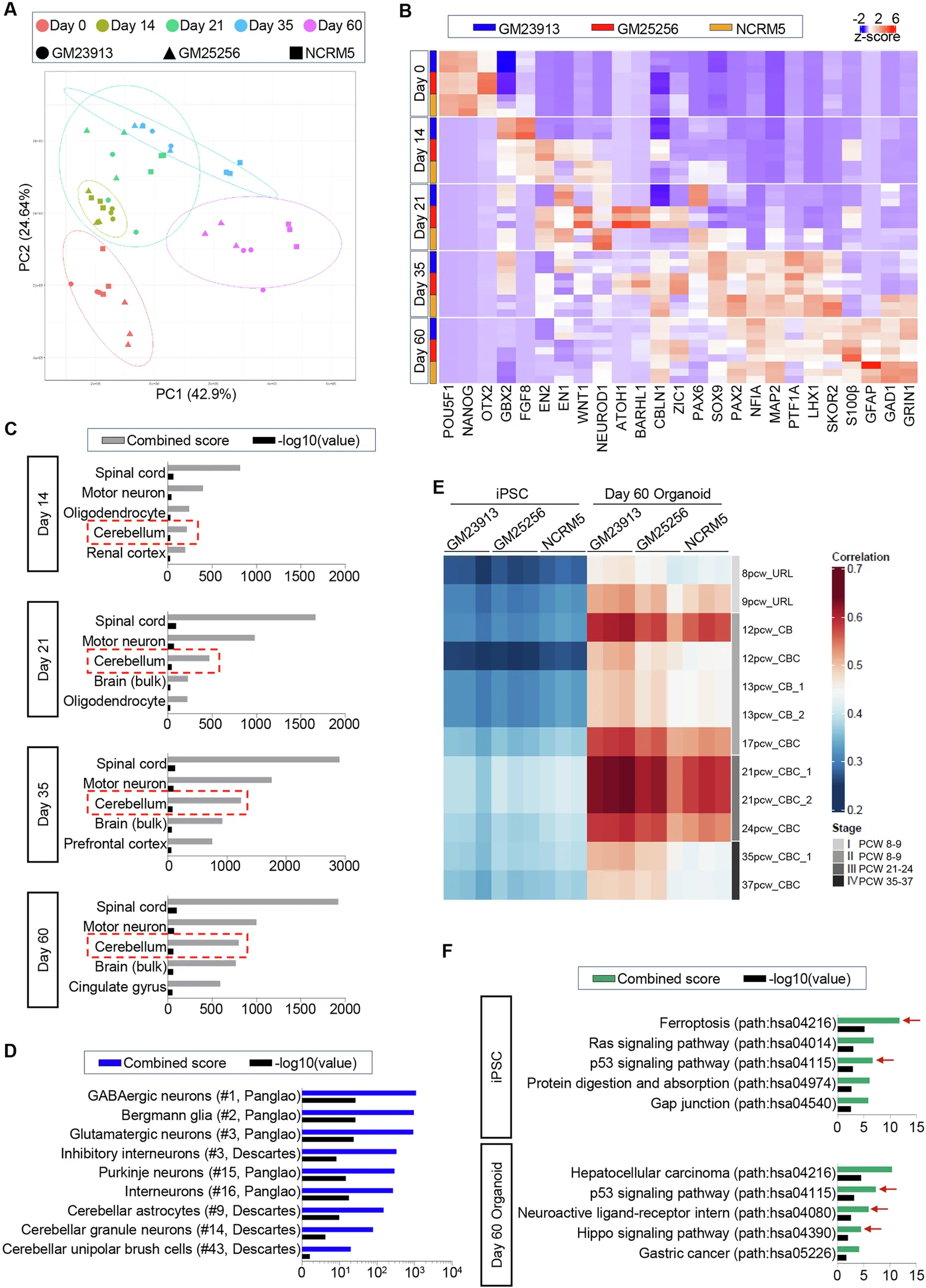
Transcriptomic analysis of hCBOs (day 60). **A** Principal component (PC) analysis plot of iPSC-derived hCBOs at different time points. Cerebellar organoids were derived from healthy donors (GM25256, NCRM5) and FRDA patient-derived (GM23913) iPSCs. **B** Heatmap analysis of key markers expressed at each time point during cerebellar organoid differentiation. *n* = 3 independent hCBO batches at each time point for three independent cell lines. **C** Analysis comparing the transcriptome of cerebellar organoids at different time points to that of human tissue samples in the ARCHS4 database. Note that cerebellum was among the top hits across the different time points. **D** Transcriptome comparison of hCBOs to previously published human cell atlases (Panglao and Descartes)^39,40^. **E** Heatmap of Spearman correlation analysis comparing day 60 hCBOs to fetal cerebellar tissues at different post-conception weeks (PCW, data from Allen BrainSpan). Each row corresponds to a distinct developmental stage of the human cerebellum. URL upper rhombic lip, CB cerebellum, CBC cerebellar cortex. **F** KEGG pathway enrichment analysis reveals distinct pathway enrichment in iPSCs and cerebellar organoids from FRDA disease (GM23913) *vs* healthy (GM25256, NCRM5) donors. Red arrows indicate metabolic pathways with relevance to FRDA pathophysiology. The top 500 differentially upregulated genes (adjusted *P*_val_ < 0.05, fold change > 2) were used in (**C**), (**D**), and (**F**).

To characterize the differentiation of iPSCs into hCBOs, we performed RNA sequencing (RNA-seq) experiments at different time points (day 0, 14, 21, 35, 60) using three different cell lines, including two from healthy individuals (GM25256, NCRM5) and one patient with FRDA (GM23913). Time-course transcriptomic analysis of differentiating hCBOs and principal component (PC) analysis demonstrated clustering of samples according to the different developmental stages (Fig. 5A), indicating reproducibility of the method and successful generation of hCBOs from healthy and patient-derived iPSC lines. Heatmap clustering analysis confirmed differentiation into R1 and cerebellar identity as measured by comparing differentially expressed (DE) genes at different time points (Fig. 5B). As expected, pluripotency-associated genes *POU5F1* (*OCT4*) and *NANOG* were expressed at day 0 and absent at day 14. Critical genes associated with the R1 region (*e.g., WNT1, FGF8, EN1, and EN2*) were expressed at day 14 and 21 (Fig. 5B). At these time points, downregulation of midbrain marker *OTX2* and upregulation of the hindbrain marker *GBX2* suggested appropriate patterning into the caudal midbrain-hindbrain boundary where R1 arises (Fig. 1B). By day 21 and 35, genes indicative of CPVZ lineage (*PAX2, SKOR2, PTF1A*) and RL (*PAX6, ATOH1, BARHL1*) were strongly expressed in the hCBOs along with typical neuronal markers (*NEUROD1, MAP2*) and gliogenesis-associated genes (*NFIA, SOX9*). In more mature hCBOs (day 60), additional genes known to be upregulated in the developing cerebellum, such as *CBLN1* (cerebellin 1 precursor), *LHX1* (transcription factor expressed by developing Purkinje cells), *GAD1* (GABA synthesis), *GRIN1* (NMDA receptor subunit), *S100β* and *GFAP* (BGC markers) were highly expressed (Fig. 5B).

To further confirm the transcriptomic signature of hCBOs, we performed unbiased gene enrichment analysis comparing the top 500 DE genes expressed by hCBOs to a publicly available tissue database containing 84,863 different human transcriptomes^38^. This analysis underscored the transcriptomic signature of our organoids and “cerebellum” was a top-ranked category from day 14–60 (Fig. 5C). Furthermore, we confirmed cerebellar cell type identities in hCBOs (day 60) by comparing the top DE genes with transcriptomic datasets from two large cellular databases, Panglao (>1 million cells analyzed from 74 tissues) and Descartes (>4 million cells analyzed from 15 tissues)^39,40^. Again, this unbiased approach showed high correlations to various neuronal and glial cell types of the cerebellum (Fig. 5D). The top categories included GABAergic/Glutamatergic neurons, Bergmann glia, and Purkinje neurons, along with other cerebellar cell types, such as interneurons, granule neurons, and unipolar brush cells (Fig. 5D). Next, to assess the maturity of hCBOs (day 60) relative to the developing cerebellum in the fetal brain, transcriptomic profiles were compared to publicly available datasets spanning from post-conception week (PCW) 8 to 37^41^. This analysis revealed that hCBOs at day 60 were comparable to the fetal cerebellum at PCW 21–24 (Fig. 5E). iPSC lines from distinct genetic backgrounds can exhibit inherent variability in differentiation potential and time-course, which may explain the divergence observed at certain time points. Nevertheless, the overall transcriptomic trajectories were consistent with robust cerebellar differentiation across all lines (Fig. 5A-D).

Next, we integrated and compared the transcriptomic profiles of hCBOs generated with our protocol (SCTL) with two previously published datasets (Fig. S6A)^13,42^. Using transcriptomic analyses focused on age-matched organoids (day 60–72), we performed unbiased gene set scoring across curated signatures (Supplementary Table 1) for forebrain, midbrain, cerebellum, and Bergmann glia. Our analysis showed that, while cerebellar signatures were comparable across studies, previously reported protocols produced cells with statistically significant higher expression levels of forebrain markers including *FOXG1, CUX2, EMX2* when compared to our dataset (Fig. S6B, C). Furthermore, this comparison revealed that hCBOs generated with our method showed greater enrichment of Bergmann glia-associated genes (Fig. S6B) and stronger expression of *GFAP, HOPX*, and *GPR37L* (Fig. S6C).

Lastly, using our transcriptomic data, we asked if disease-associated changes could be detected in hCBOs from a FRDA patient (GM23913) when compared to the healthy controls (GM25256, NCRM5) (Fig. 5F). Interestingly, gene set enrichment analysis showed upregulation in the p53 signaling pathway in FRDA samples in undifferentiated iPSCs as well as hCBOs (day 60). Upregulation of the p53 pathway was previously reported in the peripheral blood samples from 28 FRDA patients^43^. Gene enrichment analysis also shed light on cancer pathways, ferroptosis, and Hippo signaling.

### Using hCBOs from FRDA patients to identify disease phenotypes

**Fig. 6:**
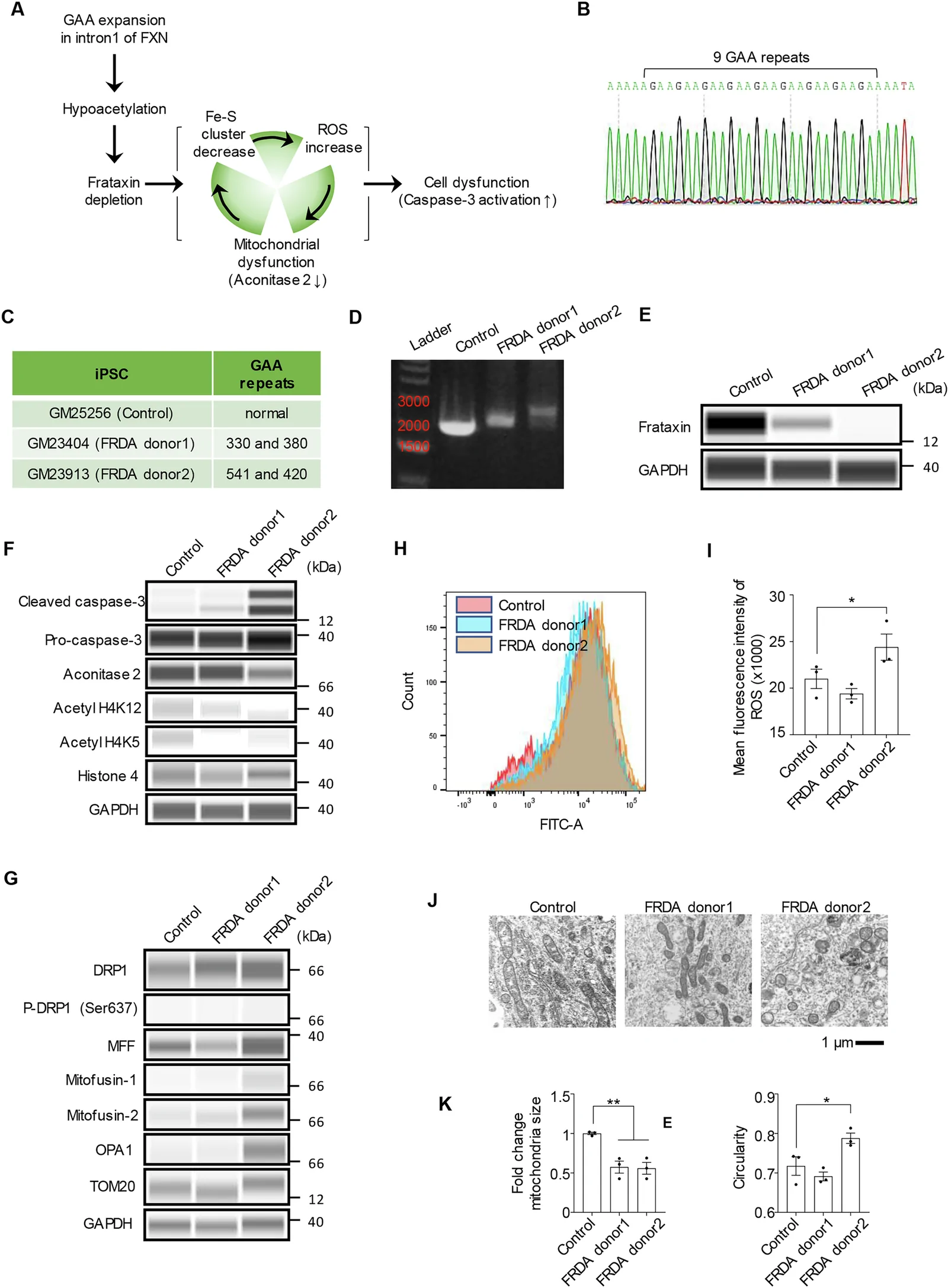
Generation of FRDA iPSC lines and identification of disease-specific phenotypes. **A** Schematic overview of FRDA pathology. Frataxin depletion results in impaired Fe-S (iron-sulfur) cluster synthesis and leads to increased ROS and mitochondrial dysfunction, ultimately resulting in cell death. **B** Sanger sequencing chromatogram showing GAA repeats within normal range in the frataxin intron 1 region of GM25256 healthy iPSCs. **C** GAA repeat expansion lengths reported in frataxin intron 1 locus of control (GM25256) and FRDA donors 1 and 2 (GM23404 and GM23913) parental fibroblasts. **D** PCR genomic analysis of the frataxin intron 1, confirming longer GAA expansion mutations in FRDA donor iPSCs. **E** Western blot demonstrating that decreased frataxin expression correlates with longer expansion mutations in FRDA iPSCs. **F** FRDA patient-derived hCBOs (day 60) exhibit increased levels of cleaved caspase-3, decreased mitochondrial enzyme aconitase 2, and hypo-acetylation of histone H4. Note that hCBOs with longer GAA repeats show more pronounced cellular pathology, which is known to correlate with clinical disease severity. **G** Western blot analysis of mitochondrial proteins in hCBOs (day 60). Key proteins involved in mitochondrial fission (DRP1, p-DRP1, MFF), fusion (OPA1), and protein import (TOM20) were analyzed in control and FRDA donor lines. **H**, **I** Quantification of intracellular ROS using fluorescence H2DCFDA demonstrates statistically significant elevation of ROS in day 60 FRDA hCBOs derived from the FRDA donor 2 with the longer expansion mutation. *n* = 3 biological replicates per group, *p < 0.05. **J**, **K** Electron microscopy images and quantification of mitochondrial size and circularity showing abnormal mitochondria in FRDA hCBOs (day 60). *n* = 3 images per group, *p < 0.05, **p < 0.01.

In FRDA, hyperexpansion of GAA repeats within the intron 1 region of the FXN locus induces repressive heterochromatin that is accompanied by hypoacetylation of histones H3 and H4^44^. This hypoacetylation contributes to transcriptional silencing of the FXN gene leading to frataxin protein depletion within cells. Frataxin plays an important role in mitochondrial function as it controls iron-sulfur (Fe-S) cluster biogenesis within the mitochondria. Impairment of the iron-sulfur cluster machinery due to a depletion in frataxin leads to an increase in reactive oxygen species (ROS), followed by mitochondria dysfunction, decreased levels of the mitochondrial enzyme aconitase 2, and eventually caspase-3 activation and cell death (Fig. 6A)^45^.

To demonstrate how hCBOs can be used for disease modeling, we performed a series of experiments that compared two FRDA patient-derived iPSC lines (FRDA donor 1 (GM23404) and FRDA donor 2 (GM23913)) to a healthy control (control (GM25256)). The iPSCs from both healthy and FRDA patients showed typical morphologies and expression of markers (OCT4, NANOG, and SOX2) associated with pluripotency (Fig. S7A). Normal FXN alleles are known to have 6–34 GAA repeats^16^, which was confirmed by Sanger sequencing in the control iPSC line (9 GAA repeats, Fig. 6B). Meanwhile, cell lines from FRDA patients had either 330/380 (FRDA donor 1) or 541/420 (FRDA donor 2) GAA repeats, respectively (Fig. 6C). Furthermore, polymerase chain reaction (PCR) analysis with primers flanking the intron 1 region of FXN generated larger fragments in the FRDA iPSCs, confirming the presence of expansion mutations in FRDA donor 1 and FRDA donor 2 (Fig. 6D). It is well-known that the length of the expansion mutation positively correlates with the extent of the FXN repression^45^. Accordingly, in comparison to the control, patient-derived iPSCs with 330/380 GAA repeats (FRDA donor 1) had a reduction of FXN expression, while the iPSC line with 541/420 GAA repeats (FRDA donor 2) was completely devoid of FXN (Fig. 6E).

Next, hCBOs were successfully generated from these three cell lines and expression of typical markers was confirmed by immunohistochemistry and Western blot (Fig. S7B, S7C). These organoids were composed of cells expressing typical markers of the CPNE lineage (GBX2, PAX2, SKOR2) and RL markers (PAX6, BARHL1) at day 35 (Fig. S7B, S7C). When hCBOs were analyzed at day 60 by Western blot, various FRDA-associated disease phenotypes were detected including increased levels of activated/cleaved caspase 3, decreased level of aconitase 2, and hypoacetylation of histone H4 (H4K5, H4K12) (Fig. 6F). Of note, disease phenotypes were most severe in hCBOs from the patient with longer GAA expansion mutation (FRDA donor 2) as compared to healthy control and FRDA with a shorter expansion mutation (FRDA donor 1). The same trend and correlation with disease severity was observed when hCBOs were analyzed for expression of mitochondrial proteins implicated in mitochondrial function and plasticity (*i.e*., fission and fusion; Fig. 6G) and ROS levels using flow cytometry (Fig. 6H, I). Lastly, ultrastructural analysis confirmed aberrant mitochondrial morphologies and differences in mitochondria size, which were more pronounced in the hCBOs generated from the iPSC line with longer GAA expansion mutations (Figs. 6J, K, S8).

### Correction of FRDA disease phenotypes

**Fig. 7:**
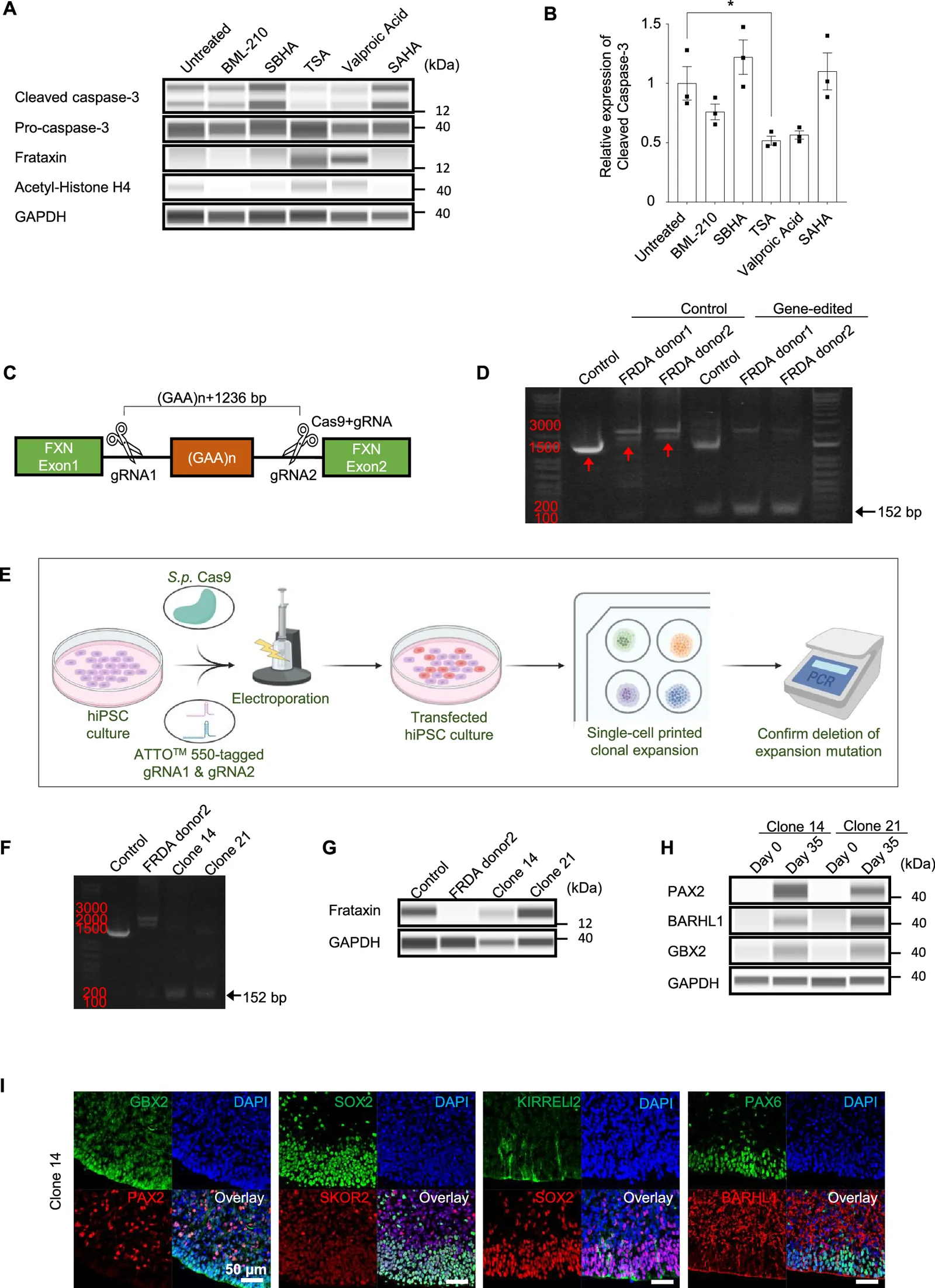
Drug testing in hCBOs from FRDA patients and gene correction by using CRISPR-Cas9. **A** Effect of HDAC inhibitors on hCBOs (day 60) from FRDA donor 2. SBHA: Suberohydroxamic acid. TSA: Trichostatin A. SAHA: Suberoylanilide hydroxamic acid. **B** Quantification of Western blot analysis performed in **A**. *n* = 3. Error bar, mean ± SEM, *p < 0.05. **C** Gene editing strategy to correct the mutation in FRDA patient-derived iPSCs by using CRISPR-Cas9. gRNA: guide RNA. **D** PCR analysis confirmed gene deletion in all the iPSC lines. The expected PCR product size post-gene editing is 152 bp. **E** Schematic overview depicting the strategy for generating clonal gene-corrected iPSC lines. The schematic diagram was created in BioRender. Tristan, C. (2026) https://BioRender.com/p0iru3r. **F** PCR analysis confirmed gene deletion in the established cell clones of corrected FRDA donor 2 iPSCs. **G** Western blot analysis of FXN expression in healthy control, FRDA donor 2, and two gene-corrected isogenic lines from FRDA donor 2 (clone 14 and 21). **H**, **I** Western blot and immunohistochemical analysis (clone 14) of hCBOs (day 35) derived from gene-corrected isogenic FRDA donor 2.

Having identified disease phenotypes characteristic of FRDA, we asked if it might be possible to correct these abnormalities. Previous studies tested whether frataxin expression could be induced in either FRDA patient-derived lymphocytes or iPSC-derived neurons in a 2D monolayer culture using HDAC inhibitors^46,47^. Using cerebellar organoids as the most relevant model for FRDA, we examined five commercially available HDAC inhibitors, testing three iPSC-derived hCBOs per drug (Fig. 7A, B). Among the tested compounds, trichostatin A (TSA) and valproic acid showed the strongest effects on inducing frataxin protein as measured by Western blot analysis. We also noted increased levels of H4 acetylation and marked reduction in activated caspase 3 (Fig. 7A, B). While HDAC inhibitors may have some beneficial effects, their long-term efficacy and safety for treating FRDA pose clinical challenges. For instance, the anti-epileptic drugs valproic acid, phenytoin, and others can damage the cerebellum and lead to the loss of Purkinje cells and GCs^48–50^. Cell and gene therapies have emerged as therapeutic options as iPSCs and gene editing technologies have entered the clinical stage, while organoids may serve as physiologically-relevant preclinical models for genetic diseases^51^. Establishment of gene-corrected isogenic iPSC lines using CRISPR-Cas9 has become an important strategy. Therefore, we employed a CRISPR-Cas9 gene-editing system to excise the GAA expansion mutation in the FXN intron 1 region of FRDA patient-derived iPSC lines (Fig. 7C) following a previous report^45^. We confirmed by using PCR gel electrophoresis that the guide RNAs (gRNAs) resulted in effective excision of the GAA mutated region as shown through shorter bands detected at 152 bp (Fig. 7D). To establish gene-corrected clonal iPSC lines, we used a single-cell printer and our previously established protocol, including the CEPT small molecule cocktail to identify and expand new cell lines (Fig. 7E)^18,52^. Two clonal iPSC lines (clone 14 and 21) from the FRDA patient with longer GAA repeats (FRDA donor 2) were successfully established and characterized (Figs. 7F, S9A, B). Long-read sequencing analysis (PacBio) verified on-target excision in the FXN intron 1 locus and the absence of off-target effects within the top four gRNA homology sites for gRNA1 and gRNA2 (Figs. S9C, D, S10). Moreover, optical genomic mapping of the genomes confirmed normal karyotypes in the established clonal lines (Fig. S11). Notably, increased frataxin expression was obtained in the isogenic gene-corrected FRDA iPSC lines (Fig. 7G). By day 35, hCBOs showed normal development and differentiation into different cell types expressing typical markers as analyzed by Western blotting and immunohistochemistry (Fig. 7H, I).

## Discussion

Here, we established an advanced hCBO model that recapitulates key aspects of orchestrated cerebellar development, including R1 specification, cerebellar plate formation, induction of BGCs, neuronal migration, and derivation of the cellular diversity of the cerebellum. Considering previous reports studying cerebellar organoids^10,13–15,53^, to the best of our knowledge, this study is the first to demonstrate the generation of hCBOs enriched with functional BGCs that resulted in enhanced electrophysiological activity and more extensive outside-in migration of GCs. Our comparative transcriptomic analysis using RNA-seq confirmed stepwise cerebellar differentiation and suggested that the molecular signature of hCBOs (day 60) was comparable to the fetal cerebellum at PCW 21-24.

Our data demonstrated the generation of R1-like cellular identity by using controlled WNT pathway activation. Similar to in vivo development, our approach promoted the expression of R1 markers (EN2, FGF8, WNT1), while hindbrain markers such as HOXA2 were not induced. In addition, utilizing our previously established combination of glial-inducing factors^29^, we efficiently generated BGCs in hCBOs produced with this 60-day protocol. This approach underscores the fact that defining optimal concentrations and combinations of small molecules and growth factors, and their administration at specific time points, are critical for appropriate patterning and guiding cell differentiation trajectories. This might be particularly important and challenging for organoids that need to recapitulate the highly complex development of the cerebellum, while avoiding the generation of unwanted cell types of other brain regions.

Understanding the mechanisms by which BGCs contribute to cerebellar patterning and function has been a long-standing interest in neuroscience^54^. Abnormal BGC development is associated with various neurodevelopmental and genetic diseases^8,9^, yet the availability of a complex human in vitro model has been limited until now. We predict that having access to cerebellar organoids, that can be generated over the course of 60 days and produce BGCs, GCs, Purkinje cells and other relevant cell types, will be crucial for modeling and gaining a deeper understanding of cerebellar development and various diseases.

To demonstrate translational utility of hCBOs, we investigated iPSC lines from patients with FRDA. Consistent with the pathophysiology of FRDA and the known functions of the FXN gene and disease severity correlating with the length of GAA repeats, we observed altered mitochondrial morphologies, oxidative stress, and elevated caspase 3 activation. The reversal of disease phenotypes by using HDAC inhibitors and generating gene-corrected isogenic cell lines by CRISPR-Cas9 was promising. Nevertheless, future studies are needed to develop new therapies that may include cell replacement, in vivo gene editing, and other disease-modifying strategies. We envisage that many other diseases affecting the cerebellum, such as the recently described FGF14 GAA repeat expansion in late-onset cerebellar ataxia, will benefit from investigating patient-derived hCBOs to deliver on the promise of precision medicine^55^.

Our study significantly advances the use of hCBOs for fundamental research in developmental biology as well as disease modeling. However, we have not cultured hCBOs for extended periods of time, but it should be possible to extend culture period beyond 2–3 months as other groups have cultured organoids for 6–20 months^13,28^. It is likely that extended culture periods will result in increased morphological complexity and more mature cell types. This is particularly important for the generation and integration of Purkinje cells with highly complex dendritic arborizations. Elucidating the axonal projections of GCs (parallel fibers innervation of Purkinje cell dendrites), Purkinje cells (the sole output from the cerebellar cortex) and other neuronal cell types will provide new insights in microcircuitry formation in this organoid model.

In addition, while segregation between CPNE/CPVZ and the RL domains can be observed using cell type-specific markers, the emergence of RL- and CPNE-like regions in this organoid model cannot fully recapitulate all aspects of in vivo development. Specifically, this model lacks dorso-ventral and anterior-posterior molecular cues, and tangential migration in the absence of secreted factors from the pial surface. Furthermore, while our transcriptomic data support the presence of Bergmann glia populations based on established marker expression, unambiguous lineage relationships would require single-cell lineage tracing and genetic labeling in future work.

In hCBOs established from FRDA patients, we could recapitulate many disease-specific phenotypes that were previously reported using other models. Following our proof-of-principle experiments, testing a greater number of donor-derived iPSC lines will help to establish a powerful disease modeling platform to study various genotype-phenotype relationships and test new therapeutic modalities.

## Methods

### Cell culture and organoid formation

Human induced pluripotent stem cells (iPSCs) were obtained from NIGMS Human Genetic Cell Repository at the Coriell Institute for Medical Research (GM25256, GM23404, GM23903) and NIH Common Fund (NCRM5). Human embryonic stem cells (hESCs) were obtained from WiCell Research Institute (WA09). Human pluripotent stem cells (hPSCs) were maintained in feeder-free conditions using mTeSR^TM^ Plus (STEMCELL Technologies) and vitronectin (VN)-coated plates (Thermo Fisher Scientific). Cell lines were confirmed to be karyotypically normal and mycoplasma-free (MycoAlert Detection Kit, Lonza). Cells were routinely passaged when cultures reached 70–90% confluency (every 3–4 days) using 0.5 mM EDTA diluted in phosphate-buffered saline (PBS) without calcium or magnesium (Thermo Fisher Scientific). Cell cultures were maintained at 37 °C under humidified 5% CO_2_ and atmospheric O_2_.

To generate human cerebellar organoids (hCBOs), hPSCs were detached using Accutase (Thermo Fisher Scientific) and plated in 24-well AggreWell^TM^ 800 plates (STEMCELL Technologies) at a density of 3000–6000 cells to form aggregates in 2 mL/well of growth factor-free chemically defined media (gfCDM), which is modified from a previous study^10^ consisting of IMDM/F12 (Gibco), chemically defined lipid concentrate (1% v/v, Life Technologies), monothioglycerol (450 µM, Sigma), apo-transferrin (15 µg/ml, Sigma), bovine serum albumin (5 mg/ml, Sigma), and supplemented with insulin (7 µg/ml, Sigma). CEPT cocktail (50 nM chroman 1 (MedChem Express), 5 µM emricasan (Selleckchem), polyamine supplement (Sigma), and 0.7 µM trans-ISRIB (Tocris)) was used in the first 2 days of cell aggregation. A83-01 (2 µM, Tocris), and LDN-193189 (100 nM, Tocris) were added on day 0^18,19^. CHIR99021 (0.5 µM, Tocris) and recombinant human FGF2 (50 ng/ml, R&D Systems) were added on day 2. The entire medium was replaced on day 2 and every other day with gfCDM supplemented with A83-01, LDN-193189, CHIR99021, and FGF2. On day 7, floating aggregates were transferred to ultra-low attachment 6-well plates (Corning) at one well of a 24-well plate to one well of a 6-well plate. Aggregates were cultured in 4 mL/well of gfCDM media with 2/3 of the initial amount of A83-01, LDN-193189, CHIR99021, and FGF2 supplements on day 7 and 1/3 on day 10. The plates were placed on an orbital shaker starting on day 10. From day 14 to day 21, media was replaced with gfCDM supplemented with recombinant human FGF19 (100 ng/ml, R&D Systems). From day 14 onward, the entire medium was replaced every 3–4 days. From day 21 to day 35, the formed organoids were cultured in DMEM/F12 GlutaMAX™ (Gibco), chemically defined lipid concentrate (1% v/v, Life Technologies), N2 supplement (Gibco), and B27 minus vitamin A (Gibco) as the base media with the addition of glial induction factors (consisting of Delta-like protein-1 (DLL1), Jagged-1, LIF, Oncostatin M, and ciliary neurotrophic factor 1 (CNTF), 10 ng/mL each, R&D Systems)^29^. Recombinant human SDF1α (300 ng/ml, R&D Systems) was added to the culture from day 28 to day 35. From day 35 onward, the media was replaced with final maturation media that consisted of DMEM/F12 GlutaMAX™ (Gibco), chemically defined lipid concentrate (1% v/v, Life Technologies), N2 supplement (Gibco), B27 (Gibco), recombinant human BDNF (50 ng/mL, R&D Systems), GDNF (50 ng/mL, R&D Systems), NT3 (50 ng/mL, R&D Systems), CNTF (10 ng/mL, R&D Systems), and T3 (1 µM, Tocris). The medium was replaced every 3–4 days. For the CHIR99021 dose-response studies, hCBOs were cultured using the same protocol up to day 14, but with various CHIR99021 concentrations at either 0, 0.5, 1, or 2 µM starting at day 2. At day 14, organoids were analyzed by Western blot.

To capture dendritic arborization of Purkinje cells, organoids were dissociated at day 60 using an Embryoid Body Dissociation Kit (Miltenyi Biotech) and gentleMACS Dissociator (Miltenyi Biotech) in a gentleMACS C Tube (Miltenyi Biotech) following the manufacturer’s protocol. The cell suspension was filtered through a 70-μm strainer (Miltenyi Biotech) to remove cell debris and clumps. The strained cell suspension was centrifuged at 300 *g* for 5 min and plated on wells coated with poly-L-ornithine (PLO) (15 μg/mL, Sigma) and laminin (20 μg/mL, Sigma), at a seeding density of 100,000 cells/cm^2^. Cells were cultured with the maturation media, which was replaced every 3–4 days. 30 days after re-plating, cells were fixed in 4% paraformaldehyde (PFA; Thermo Fisher Scientific) for 20 min at RT and washed three times with PBS.

To generate cortical organoids, we implemented a modified method from a previously published protocol^27^. iPSCs were dissociated into single cells using Accutase (Thermo Fisher Scientific) and plated in 96-well ULA round-bottom plates (Corning) at a density of 6000 cells per well with the CEPT cocktail in cortical differentiation medium (CDM) I, containing Glasgow-MEM (Gibco), 20% Knockout Serum Replacement (Gibco), 0.1 mM Minimum Essential Medium non-essential amino acids (Gibco), 1 mM sodium pyruvate (Gibco), and 0.1 mM 2-mercaptoethanol (Gibco). TGF-β inhibitor SB431542 (Tocris) and WNT inhibitor IWR-1 (Tocris) were added at a concentration of 5 µM and 3 µM, respectively. Media was changed every 3 days with CDM1 48 h after seeding. After 18 days, the aggregates were cultured in 100-mm ULA culture dishes (Corning) on an orbital shaker and media was changed to CDM II containing DMEM/F12 medium (Gibco), 2 mM GlutaMAX™ (Gibco), 1% N2 supplement (Gibco), and 1% chemically defined lipid concentrate (Gibco). The media was changed every 3 days until day 35.

### Western blot analysis

Organoids were washed in PBS, resuspended in RIPA buffer (Thermo Fisher Scientific) supplemented with Halt protease inhibitor cocktail (Thermo Fisher Scientific) and lysed by sonication. Cell debris was removed by centrifugation at 14,000 *g* for 15 min. Protein quantification was performed using the BCA protein assay kit (Thermo Fisher Scientific). The Wes and Jess automated Western blotting systems (ProteinSimple) were utilized following the manufacturer’s instructions. All Western blot data are presented by lanes in virtual blot-like images. For Western blot quantification, band intensity was calculated using ImageJ. The relative amount of the target protein was normalized to GAPDH. Detailed information on primary and secondary antibodies is provided in Supplementary Table 2.

### BrdU analysis

hCBOs were pulse-labeled with 10 µM BrdU for 2 h at 37 °C, followed by three washes with PBS, at either day 28 or day 32 to assess migratory behavior. At day 35, hCBOs were fixed in 4% PFA, cryoprotected in 30% sucrose, embedded in OCT, and sectioned at 10 µm. Sections were permeabilized with 0.3% Triton X-100, 5% donkey serum, and 1% BSA for 1 h at room temperature, followed by DNA denaturation in 2 N HCl at 37 °C for 1 h. After three washes in permeabilization buffer, sections were immunostained with anti-BrdU and TUJ1 overnight at 4 °C. Appropriate secondary antibodies were applied for 45 min at room temperature. Details of primary and secondary antibodies are provided in Supplementary Table 2. Slides were mounted using ProLong Glass Antifade Mountant with NucBlue Stain (Thermo Fisher Scientific), and fluorescence images were captured using a Zeiss LSM 710 confocal microscope with appropriate filters. To quantitatively assess BrdU^+^/TUJ1^+^ cells, we applied an enhanced fiber-based image analysis framework. Within predefined bottom-surface regions of interest corresponding to the organoid surface, fiber-like signals were selectively enhanced using a multiscale Hessian-based ridge detector and subsequently converted into binary representations via adaptive local thresholding and morphological processing to preserve structural continuity. The resulting fiber masks were spatially intersected with surface-restricted DAPI masks to delineate TUJ1-positive neuronal objects and BrdU/TUJ1 double-positive structures within the region of interest. For each segmented object, centroid coordinates were computed, and migration distance was defined as the vertical separation between the centroid and the local bottom-surface boundary derived from the corresponding ROI mask. Distances were summarized on a per-label basis using mean statistics, providing a quantitative measure of the spatial distribution of BrdU^+^/TUJ1^+^ relative to the organoid surface. Analyses were implemented in an automated and reproducible pipeline.

### Histological analysis

Organoids were fixed in 4% PFA at RT for 3 h, followed by three washes with PBS. Subsequently, they were incubated in 30% sucrose at 4 °C overnight. Tissues were then embedded in 7.5% gelatin/10% sucrose embedding solution (Sigma), molded in dry ice/acetone solution, sectioned into 10-µm slices, and mounted onto microscope slides (Fisher Scientific) for staining. For histological analysis, sections underwent staining with H&E, dehydration in a series of ethanol concentrations (70%, 80%, 95%, 100%) and 100% xylene, stained in hematoxylin and eosin dye, and mounted on coverslips using Permount mounting medium (Fisher Scientific). For immunohistochemical analysis, sections were permeabilized and blocked with 0.3% Triton X-100, 5% donkey serum, and 1% BSA in PBS for 1 h. Detailed information regarding primary and secondary antibodies can be found in Supplementary Table 2. Slides were mounted using ProLong Glass Antifade Mountant with NucBlue Stain (Thermo Fisher Scientific), and fluorescence images were captured either using the Akoya PhenoImager HT or Zeiss LSM 710 confocal microscope with appropriate filters.

### Image-based neuronal migration analysis

NeuN^+^/GFAP^+^/DAPI^+^ and calbindin^+^/NeuN^+^/DAPI images captured using the Zeiss LSM 710 confocal microscope were analyzed using Simple Linear Iterative Clustering algorithm, a superpixel algorithm that clusters pixels based on color similarity and proximity in the image plane, and Graph Cut to quantify neuronal migration in cerebellum organoids. This approach enabled the precise measurement of neuronal identification, quantification, and migration dynamics within the organoid structure. A deep learning method in Cellpose was employed to individually segment NeuN, GFAP, calbindin, and DAPI within the hCBO. Using the superpixel machine learning algorithm, precise measurements were obtained for the positions and quantification of calbindin^+^/NeuN^+^ cells and the boundaries of the GFAP/NeuN layer. At the final stage, the results obtained in pixels were converted into µm units. To more precisely capture the structural organization of GFAP-positive radial glial fibers, we further incorporated an enhanced fiber-enhancement–based image analysis module enabling robust detection of radial glial scaffolds. Enhanced images were binarized using adaptive thresholding and morphological refinement to generate GFAP-positive fiber masks. Quantification was restricted to a single structural metric, *i.e*., area of GFAP-positive fibers, defined as the total number of pixels occupied by the binary fiber mask within the ROI. This metric provides a direct measure of the spatial coverage and structural expansion of GFAP-positive scaffolds and was used to compare glial-induced and non-induced organoids across experimental conditions. All analyses were performed in an automated and reproducible manner.

### Microelectrode array (MEA) analysis for an electrophysiology study

Neuronal activity was analyzed using the Maestro Pro multiwell microelectrode array system (Axion Biosystems) according to the manufacturer’s protocol. Briefly, day 60 hCBOs were plated on PLO/laminin-coated 48-well MEA plates following the manufacturer’s protocol with final maturation media. The neuronal activity was recorded for 5 min on day 80, which is 2–3 weeks post-plating. For ATP stimulation, the neuronal activity was recorded immediately following the addition of 100 µM ATP (Sigma).

### Sanger sequencing, PCR gel electrophoresis, and PacBio long sequencing

Human iPSCs were washed in PBS, centrifuged, and processed for genomic DNA extraction. Genomic DNA was extracted with GeneJet genomic DNA purification column kit (Thermo Fisher Scientific) according to the manufacturer’s protocol. For PCR amplification, Platinum^TM^ SuperFI II PCR Master Mix (Thermo Fisher Scientific) was used following the manufacturer’s protocol. Primer sets can be found in Supplementary Table 3. The amplified PCR samples were run on E-Gel^TM^ 1% agarose gels with SYBR^TM^ Safe DNA Gel Stain (Invitrogen) for visualization with E-Gel^TM^ sample loading buffer (Invitrogen). For Sanger sequencing, primer extension sequencing was performed by AZENTA Life Sciences using commercial dye terminators. The reactions were then run on an ABI 3730xl DNA Analyzer (Applied Biosystems). For PacBio long sequencing, the short fragments (<10 kb) of the HMW gDNA from the 5 hiPSC samples were removed using Short Read Eliminator (SRE) kit (Pacific Biosciences). 2 µg of DNA were sheared to 14–19 kb using the Megaruptor 3 (Diagenode, Inc.) at shear speed 31. The sheared DNA was purified using 1X SMRTbell cleanup beads and used to prepare whole-genome libraries using SMRTbell Prep Kit 3.0 (Pacific Biosciences). Sequencing primer was annealed and Revio polymerase was bound to the library prior to loading using the Revio Polymerase kit (Pacific Biosciences). Each library was loaded on one Revio SMRTcell at 200 pM loading concentration. Sequencing was performed with a 24 h movie time on Revio.

### ROS quantification

For ROS quantification, day 60 hCBOs were washed twice in DPBS (Thermo Fisher Scientific) and dissociated into a single-cell suspension using Embryoid Body Dissociation Kit (Miltenyi Biotech) and gentleMACS Dissociator (Miltenyi Biotech) in a gentleMACS C Tube (Miltenyi Biotech) following the manufacturer’s protocol. The cell suspension was filtered through a 70-μm strainer (Miltenyi Biotech) to remove cell debris and clumps. The strained cell suspension was centrifuged at 300 *g* for 5 min and stained with H_2_DCFDA reagent (Thermo Fisher Scientific) according to the manufacturer’s protocol. The stained cells were analyzed by flow cytometry (SONY SH800) using the FITC filter.

### Electron microscopy

Day 60 hCBOs were first fixed in 2% glutaraldehyde (v/v) in sodium cacodylate buffer (0.1 M, pH 7.4), followed by rinsing in cacodylate buffer, and post-fixation in 1% osmium tetroxide (v/v) solution (Electron Microscopy Sciences) in cacodylate buffer. After further rinsing in cacodylate buffer and acetate buffer (0.1 M, pH 4.0), the organoids underwent uranyl acetate (0.5% w/v, pH 4.5) en bloc staining. Dehydration was carried out using a series of ethanol concentrations (e.g., 35%, 50%, 75%, 95%, and 100%), followed by washing and overnight exposure to pure epoxy resin (Electron Microscopy Sciences). The next day, the organoids were embedded in fresh epoxy resin and processed further in a 55 °C oven for 48 h. The cured epoxy was separated by submerging it in liquid nitrogen. Thin sections (60–70 nm) were then prepared using a diamond knife and ultramicrotome (Leica). These sections were mounted onto 150 copper mesh grids and counter-stained with aqueous uranyl acetate (0.5% w/v) and Reynolds’ lead citrate. Finally, the samples were examined using an electron microscope (Hitachi), with images captured using a CCD camera (AMT).

### RNA bulk-sequencing analysis

RNA was extracted (three samples for each group, each sample consisting of three organoids) using the RNeasy Mini Kit (Qiagen). RNA was quantified using the Agilent RNA ScreenTape System (Agilent) on a 4200 TapeStation (Agilent). RNA (500 ng, RIN > 8) was used to prepare RNA-seq libraries with the KAPA mRNA HyperPrep Kit (Roche) according to the manufacturer’s protocol. Sequencing libraries were quantified by qPCR using the KAPA library quantification kit (Roche) using QuantStudio 12K Flex Real-Time PCR System (Thermo Fisher Scientific). All samples were normalized according to concentration and pooled. Libraries were loaded and sequenced using the Illumina Novaseq 6000 system. Bioinformatics analysis for bulk RNA-seq was carried out using the computational resources of the NIH HPC Biowulf cluster (http://hpc.nih.gov) using R language 3.6.0 (https://cran.r-project.org/). Bulk RNA-Seq samples were quality trimmed using Trimmomatic 0.36 and the TruSeq3 paired-end adapters. STAR aligner 2.7.6a followed by HTSeq-count 0.9.1 produced deduplicated counts of reads in genes. UCSC gene counts were normalized using the default median-of-ratios method in DESeq2 1.24.0. Differential expression tests used the lfcShrink function and gene set enrichment was performed using Enrichr API package enrichR 1.0. We compared data from samples collected at day 60 under our protocol with day 60 and day 72 datasets from two publicly available protocols deposited in GEO (GSE247974 and GSE233574). The GSE247974 dataset contains three replicates from one sample, and GSE233574 includes one replicate with single-cell sequencing data. To enable comparison between our bulk RNA-seq data and the public datasets, we generated pseudo-bulk RNA-seq counts from the single-cell data and performed three independent sub-samplings of cells from GSE233574. After merging our bulk RNA-seq data with the pseudo-bulk datasets, we removed genes with a total count below 10. Quality-controlled data were normalized in R using the vst function from the DESeq2 package, and principal components were computed with the prcomp function using normalized counts. Mixed-effects models were used to assess differential expressions between our protocol and each public protocol across gene sets representing different brain regions and Bergmann glial cells. To visualize gene-specific effects, we generated heatmaps of selected genes using normalized counts scaled within each gene.

### HDAC inhibitor testing on day 60 hCBOs

GM23913 hCBOs at day 60 were either untreated or treated with HDAC inhibitors at their reported IC_50_ value for HDAC inhibition, *i.e*., BML-210, SBHA, SAHA, all at 5 µM, TSA at 0.1 µM, and valproic acid at 400 µM^46^. The organoids were analyzed by Western blot after 24 h of treatment.

### Generation of corrected, isogenic FRDA hiPSC lines

CRISPR RNA pairs were designed in regions of intron 1 of the FXN gene (Gene ID:2395) with CRISPOR http://crispor.tefor.net, which has been previously reported (see Supplementary Table 4 for sequences)^45^. Next, gRNA was formed by thermal incubation of 100 µM crRNA, 100 µM ATTO^TM^ 550-tagged tracrRNA, and nuclease-free duplex buffer (all from Integrated DNA Technologies) at a ratio of 4.4:4.4:1.2 at 95 °C for 5 min followed by incubation at RT for 10 min. Next, the ribonucleoprotein complex was formed by incubating the formed gRNA with 36 µM Alt-R Cas9 nuclease (Integrated DNA Technologies) at a ratio of 1:1 in RT for 20 min. To generate corrected, isogenic hiPSC lines, hiPSCs were electroporated using the NEON Transfection system (Thermo Fisher Scientific) according to the manufacturer’s protocol. In brief, cells were dissociated into single cells with Accutase (Thermo Fisher Scientific), resuspended in Neon Resuspension Buffer to a density of 2.2 × 10^7^ cells/mL, and mixed with the formed RNP complexes at a ratio of 1 (RNP complex of gRNA1):1 (RNP complex of gRNA2):9 (cell suspension). The mixture was electroporated using 10 µl-pipette tips (1400 V, 20 ms, 1 pulse). Electroporated cells were seeded into VN-coated 24-well plates containing mTeSR^TM^ Plus supplemented with CEPT. Three days after electroporation, cells were detached with Accutase and either analyzed with PCR gel electrophoresis to confirm the designed CRISPR-Cas9 system efficacy or printed with Hana (Namocell) single-cell printer for clonal expansion following a previously published protocol^52^.

### Optical genomic mapping

Ultra-high molecular weight (UHMW) gDNA was extracted with the Bionano Prep SP-G2 Blood & Cell DNA Isolation Kit (Bionano Genomics) as described in the Bionano Prep SP Frozen Cell Pellet DNA Isolation Protocol (Bionano Genomics). Briefly, 1.5 million frozen pelleted cells were thawed in a 37 °C bath and resuspended in DNA Stabilizing Buffer (Bionano Genomics). After that, the cells were lysed in the presence of detergents, proteinase K, and RNase A. The UHMW gDNA was bound to a silica Nanobind Disk (Bionano Genomics), washed, and eluted. The extracted gDNA was equilibrated for 10 days at 4 °C to homogenize and then quantified with the Qubit dsDNA BR assay kit (Thermo Fisher Scientific). The isolated UHMW gDNA was fluorescently labeled using the Bionano Prep DLS-G2 labeling kit (Bionano Genomics, 80046) according to the Bionano Prep DLS-G2 Protocol (Bionano Genomics). In short, Direct Label Enzyme (DLE-1) and DL-green fluorophores were used to label 750 ng of purified gDNA at a specific sequence motif. After a cleanup of the fluorophores excess, DNA backbone was counterstained overnight before quantitation with Qubit dsDNA HS assay kit (Thermo Fisher Scientific). Finally, the labeled UHMW gDNA molecules were loaded on a Saphyr G3.3 chip for sequential imaging across nanochannels on the Saphyr instrument (Bionano Genomics). Genome analysis was performed using Bionano Solve v3.7 and Access v1.7.2. For genome assembly and variant analysis, Bionano De Novo assembly pipeline was run to assemble a genome from the molecule files (*bnx) for each sample with the human GRCh38 reference provided.

### Statistics and reproducibility

All results are shown as mean ± SEM. Statistical tests included unpaired, two-tailed Student’s *t* tests and one-way ANOVA for multiple comparisons with Tukey’s significant difference post hoc test using GraphPad Prism 9.0.0. A *P* value of <0.05 was considered statistically significant. *P* values denote as *p < 0.05, **p < 0.01, ***p < 0.001, or ****p < 0.0001. The sample sizes and number of replicates are stated in each figure legend.

### Reporting summary

Further information on research design is available in the Nature Portfolio Reporting Summary linked to this article.

## Supplementary information

Supplemental Material

Description of Additional Supplementary Files

Supplementary Data 1

Reporting Summary

## Data Availability

Additional data, including the uncropped and unedited blot/gel images, are provided in Supplementary Information. RNA-seq and whole genome sequencing data generated in this study can be found in the NCBI SRA under BioProject PRJNA1263825. Numerical source data for all graphs is included in Supplementary Data 1.

## Code availability

All custom code that supported the findings of this study is available at https://github.com/ncats/cerebellar-organoid-paper-code^56^.
