# Supplementary material for: Modeling Friedreich’s ataxia with Bergmann glia-enriched human cerebellar organoids
Source: Commun Biol. 2026 May 8;9:1235. doi: 10.1038/s42003-026-10180-5 (PMC13598134; doi:10.1038/s42003-026-10180-5)
Supplement: Supplementary file 1 [file 42003_2026_10180_MOESM1_ESM.pdf]

Supplementary Information

Modeling Friedreich's ataxia with Bergmann glia-enriched human cerebellar organoids

Seungmi Ryu*, Jason Inman, Hyenjong Hong, Vukasin M. Jovanovic, Qiang Chen, Yeliz Gedik, Yogita Jethmalani, Inae Hur, Majid Harouni, Ty Voss, Justin Lack, Jack Collins, Pinar Ormanoglu, Anton Simeonov, Carlos A. Tristan*, and Ilyas Singeç*

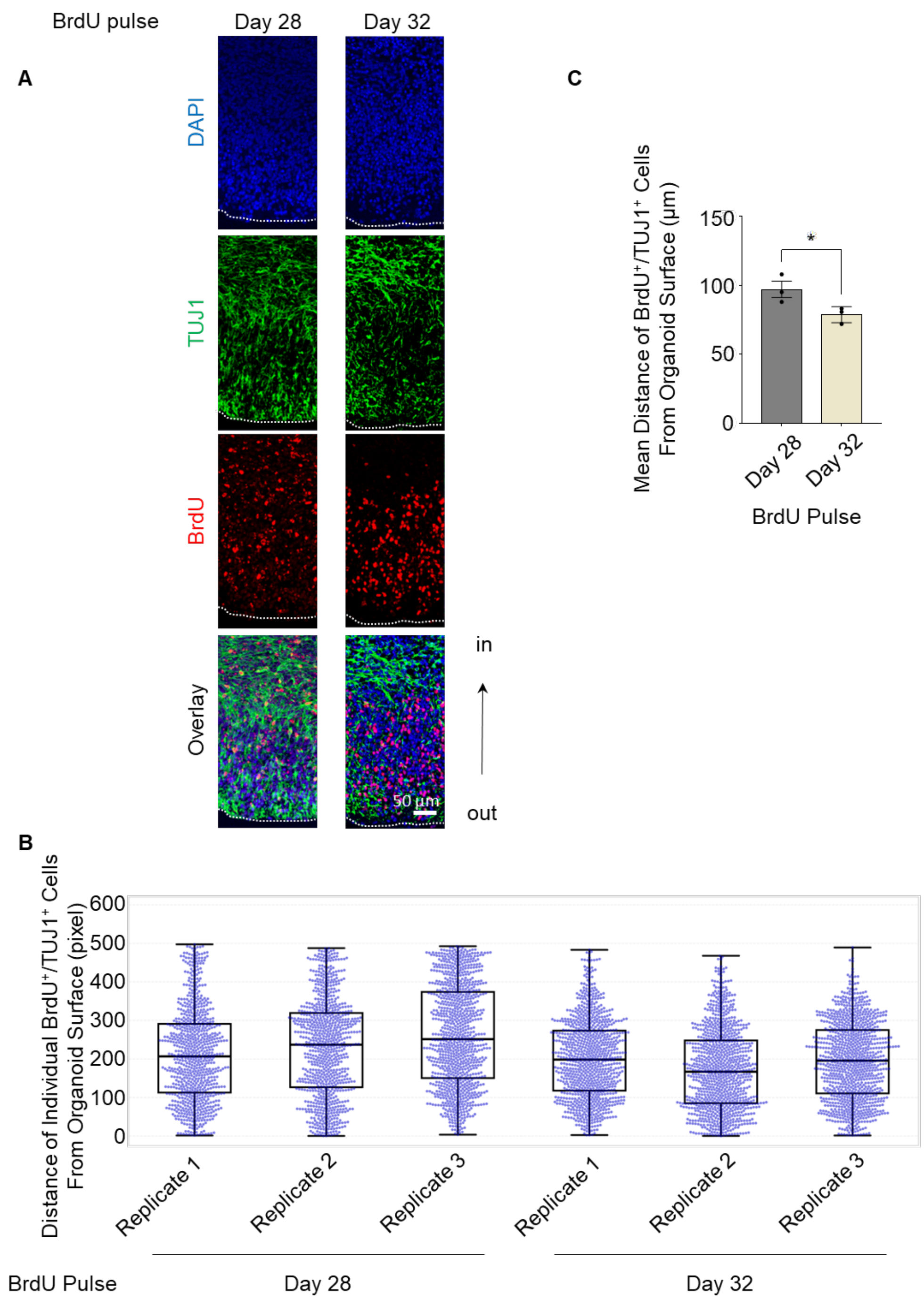

Figure S1. Birth dating analysis demonstrates outside-in neuronal migration in hCBO

(A) Representative images of BrdU and TUJ1 immunolabeling in hCBOs. A 2-hour BrdU pulse was administered either 7 days (day 28 hCBO) or 3 days (day 32 hCBO) prior to analysis at day 35. White dashed lines denote the outer surface of the hCBOs.
(B) Box-and-whisker plots showing the distance of individual BrdU/TUJ1 double-positive cells from the outer surface of the organoid. Each replicate represents an independent biological organoid.
(C) Quantification of BrdU/TUJ1 double-positive cells based on mean distance values shown in (B). 1 pixel equals 0.4151329 µm. n=3. Error bar, mean ± SEM, **p*<0.05 based on one-tailed t-test.

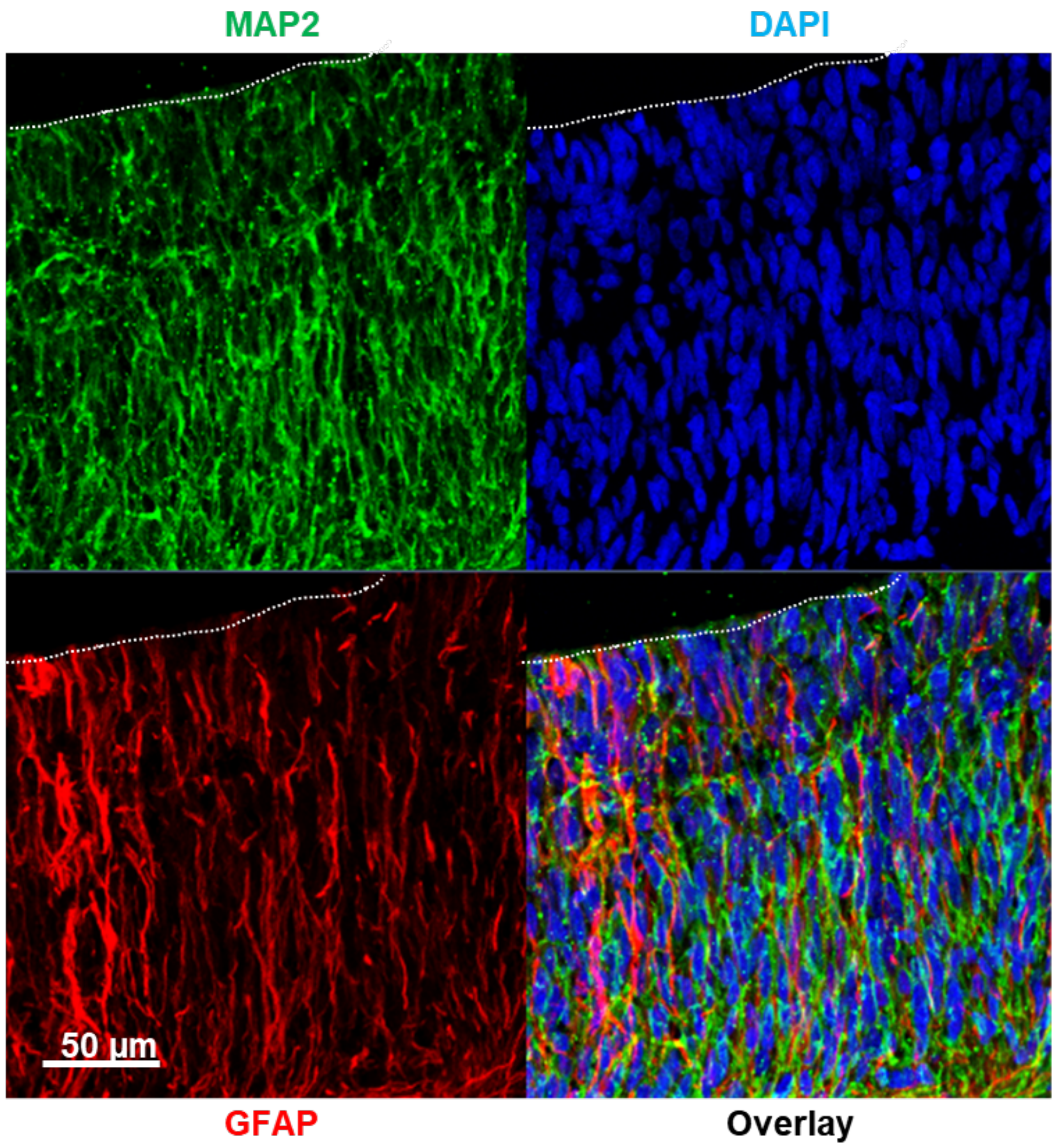

Figure S2. Immunohistochemical analysis of GFAP and MAP2 expression
Representative images of hCBO (day 60) showing radially oriented $GFAP^{+}$ fibers of BGCs providing structural guidance for neuronal migration. Neuronal structures were stained by using an antibody against MAP2. Presented images are enlarged versions of Fig. 2C.

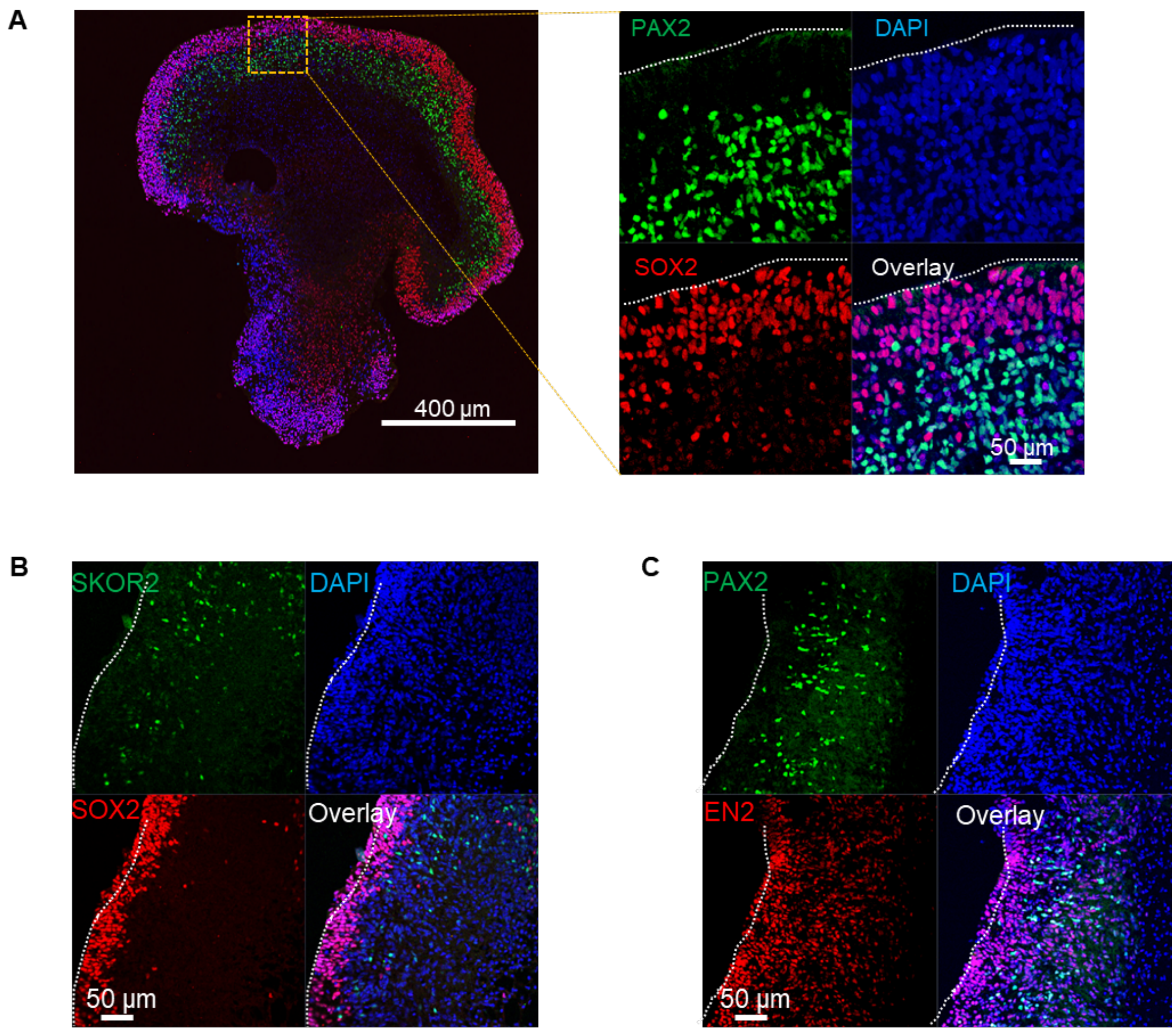

Figure S3. Immunohistochemical analysis of hCBOs (day 21)
(A) Overview image of cells expressing SOX2 or PAX2 in a histological section of a whole organoid. The image on the right depicts a higher magnification of the boxed region, illustrating distinct cell layers reminiscent of the developing cerebellum. White dashed lines indicate the outer layer of hCBOs.
(B-C) Expression of typical markers of early cerebellar development. Note the specific localization of cells expressing SOX2, EN2, SKOR2 and PAX2.

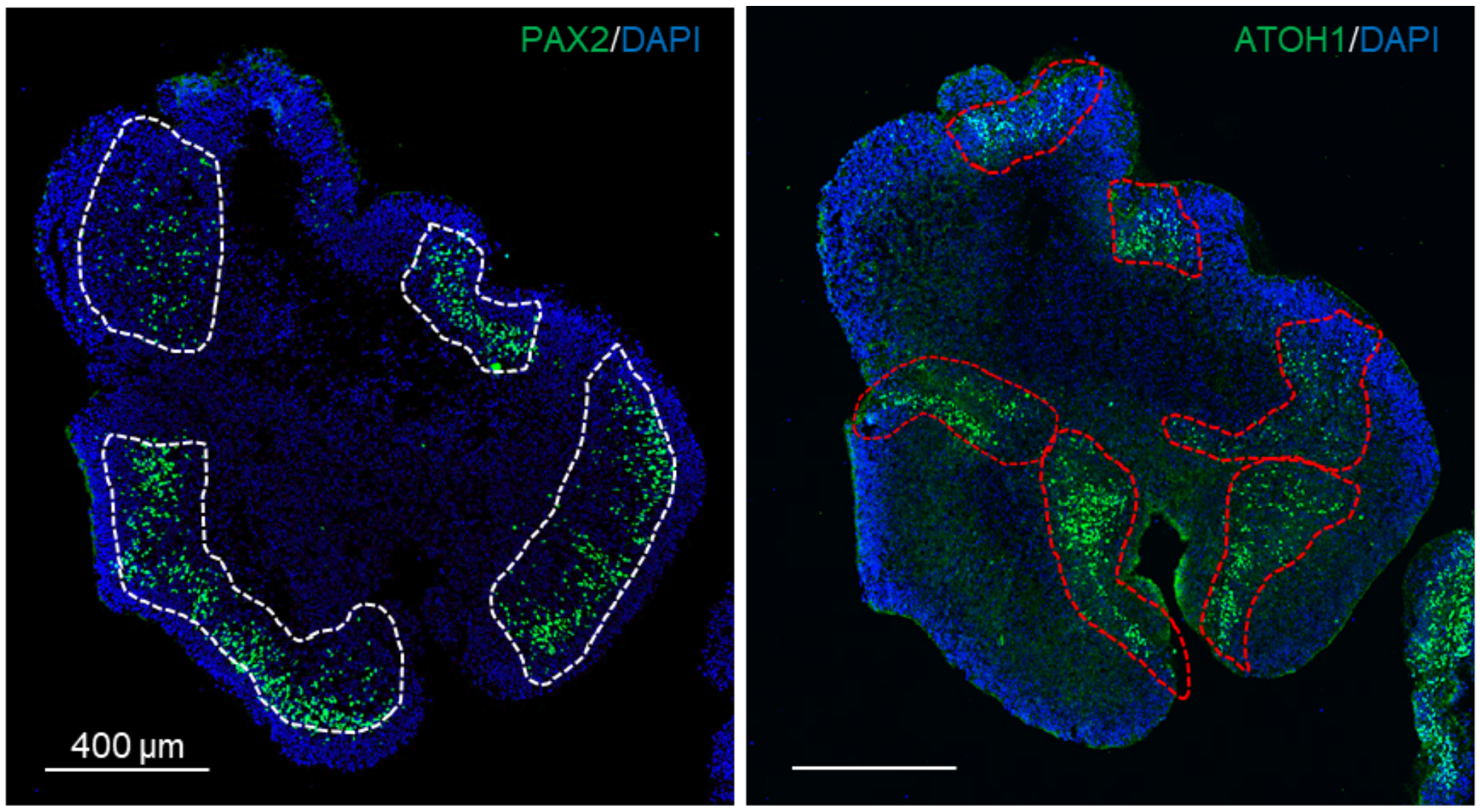

Figure S4. Distribution of CPNE- vs RL-like regions within hCBOs
Representative images showing the distribution of cells representing the CPNE (PAX2, white dashed lines) and RL lineages (ATOH1, red dashed lines) in consecutive 10 µm-thick histological sections of the same organoid (day 21).

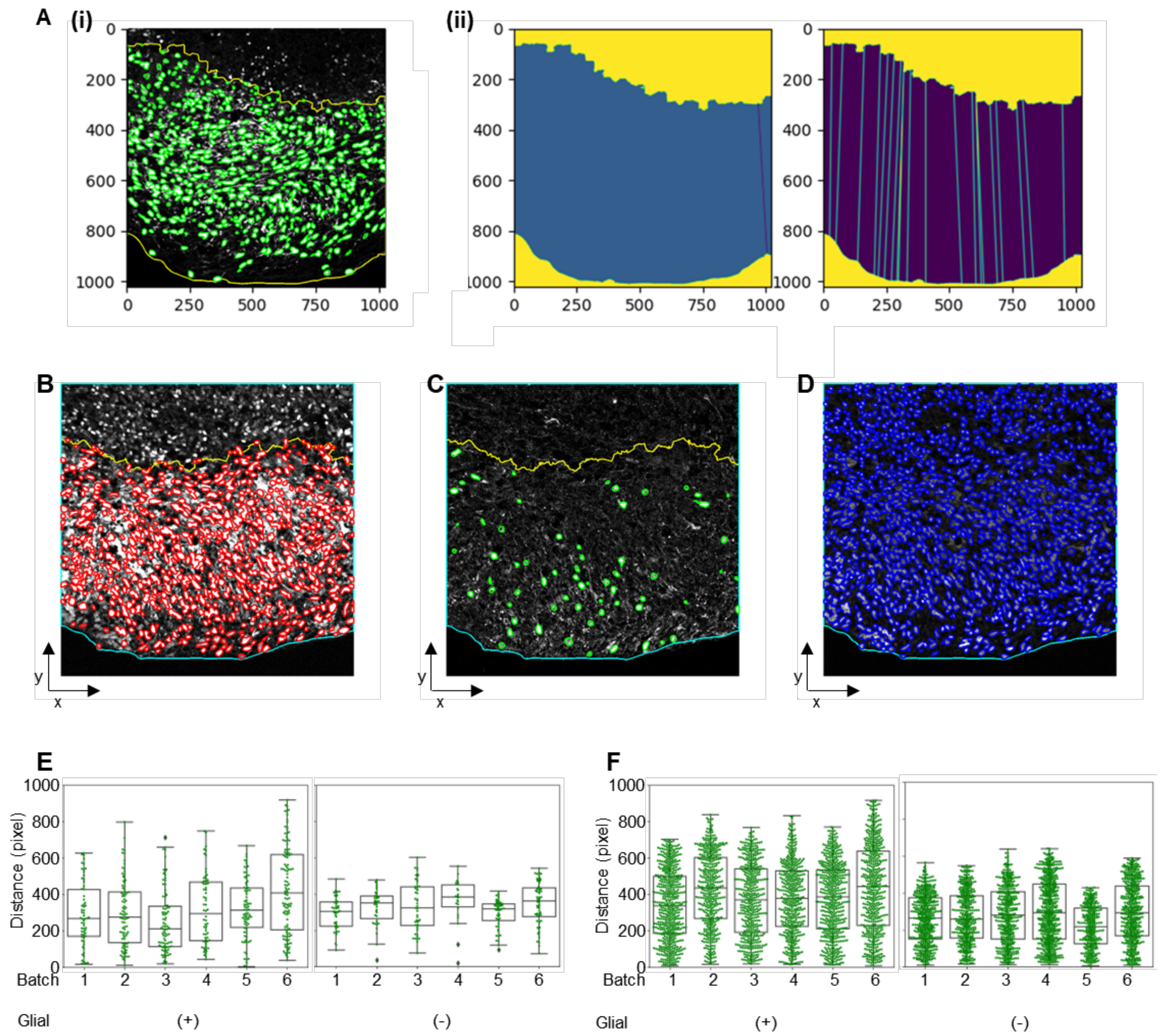

Figure S5. Machine learning-based image analysis to track neuronal migration

(A) Image-based analysis of the size of the cell layer containing NeuN-expressing neurons. The NeuN$^+$ GCs (masked green in (i)) were identified using an optimized CellPose deep learning method. Upper and lower length regions containing NeuN$^+$ cells were defined by the Super Pixel$^+$ Graph Cuts machine learning method and highlighted with yellow lines in (i) and quantified in (ii).

(B-D) Using the same method as in (A), NeuN (highlighted red in (B)) and calbindin (highlighted green in (C)), DAPI (highlighted blue in (D)), and outer surface of the organoid (highlighted cyan in (B)-(D)) were identified.

(E-F) Box and whisker plots showing (E) the distance of individual calbindin$^+$ Purkinje cell and (F) the distance of NeuN$^+$ GCs from the organoid surface with and without glial induction.

The unit is shown in pixels. 1 pixel equals 0.4151329 μm in (A), (E), and (F).

A

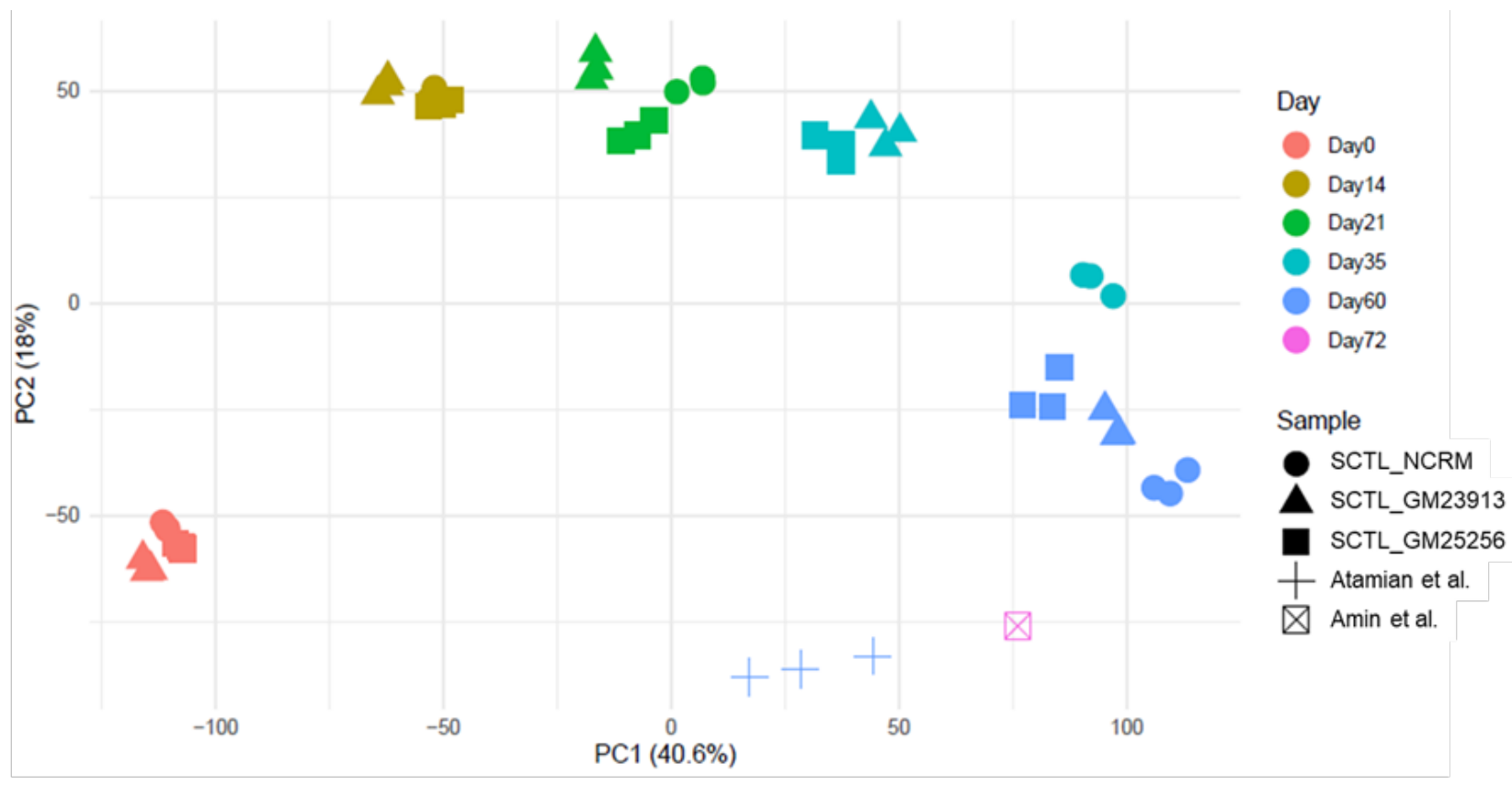

B

Comparison	Region	P value	FDR	Avg_SCTL	Avg_Comparison
SCTL_ALL_Day60vs_Amin *et al*.	Forebrain	0.00868	0.026	655.36	1,015.98
SCTL_ALL_Day60vs_Amin et *al.*	Bergmann glia	6.41E-07	0.00000353	3,791.13	929.38
SCTL_ALL_Day60vs_Amin et *al.*	Midbrain	0.102	0.102	650.59	841.22
SCTL_ALL_Day60vs_Amin et *al.*	Cerebellum	0.0842	0.102	1,234.26	1,551.13
SCTL_ALL_Day60vs_Atamian *et al.*	Forebrain	0.00135	0.00406	655.36	8,506.85
SCTL_ALL_Day60vs_Atamian *et al.*	Bergmann glia	0.000572	0.000997	3,791.13	2,048.60
SCTL_ALL_Day60vs_Atamian *et al.*	Midbrain	0.201	0.201	650.59	504.05
SCTL_ALL_Day60vs_Atamian *et al.*	Cerebellum	0.0843	0.126	1,234.26	1,516.56

C

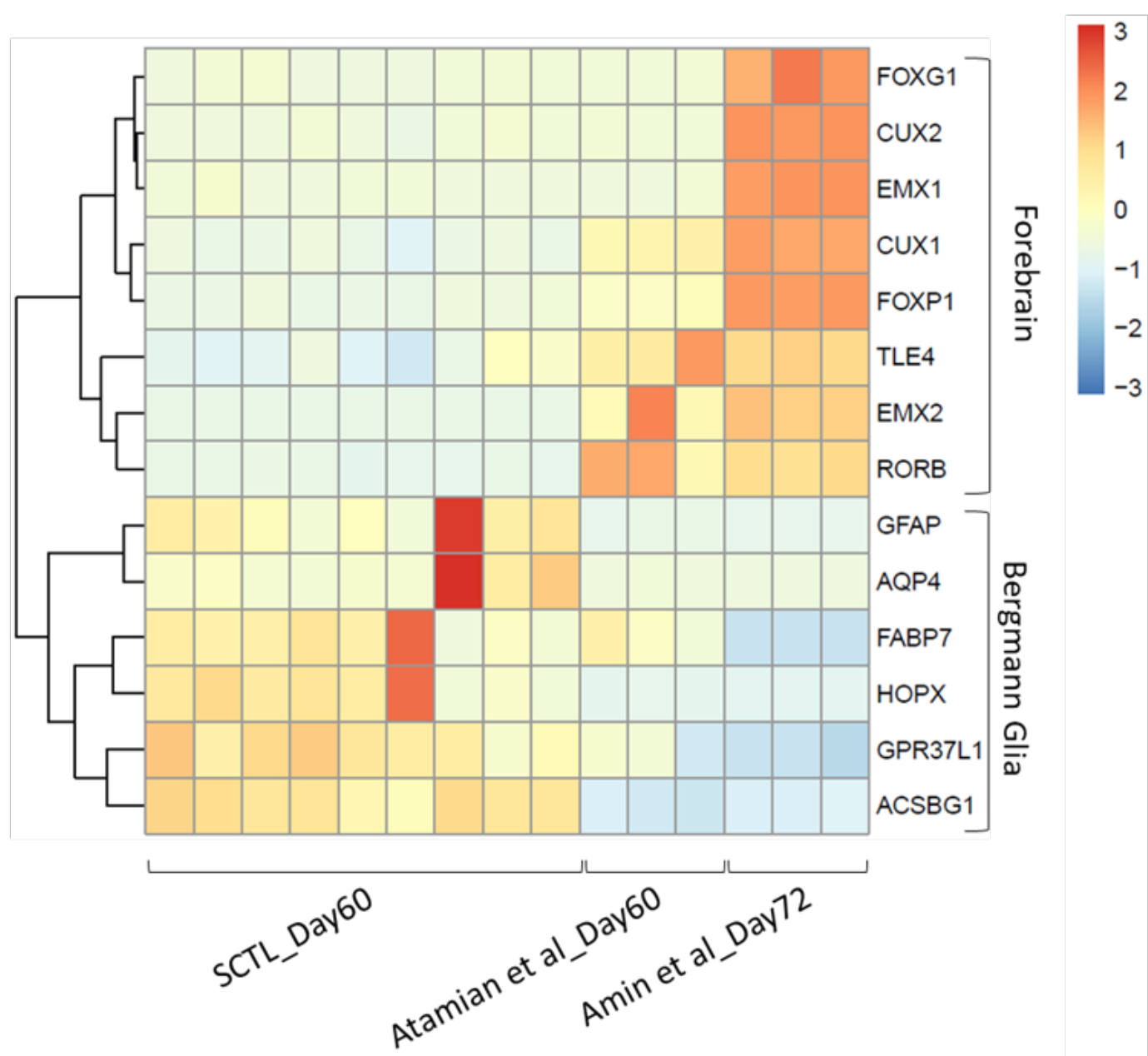

Figure S6. Transcriptomic analysis comparing iPSC-derived cerebellar organoids from the present study to previously published datasets

(A) Principal component analysis plot of iPSC-derived hCBO profiles integrated from three different studies, including the present study from the Stem Cell Translation Laboratory (SCTL), Atamian *et al.*[1], and Amin *et al.*[2]. Pseudo-bulk RNA-seq counts on single-cell RNA-seq data from previous studies were performed. The dataset published by Amin *et al.* underwent three sub-samplings for statistical comparison.

(B) Gene set scoring of age-matched organoids (day 60-72) based on curated molecular signatures for forebrain, midbrain, cerebellum, and Bergmann glia.

(C) Heatmap analysis of forebrain and BGC markers show differences in hCBOs generated in three different studies.

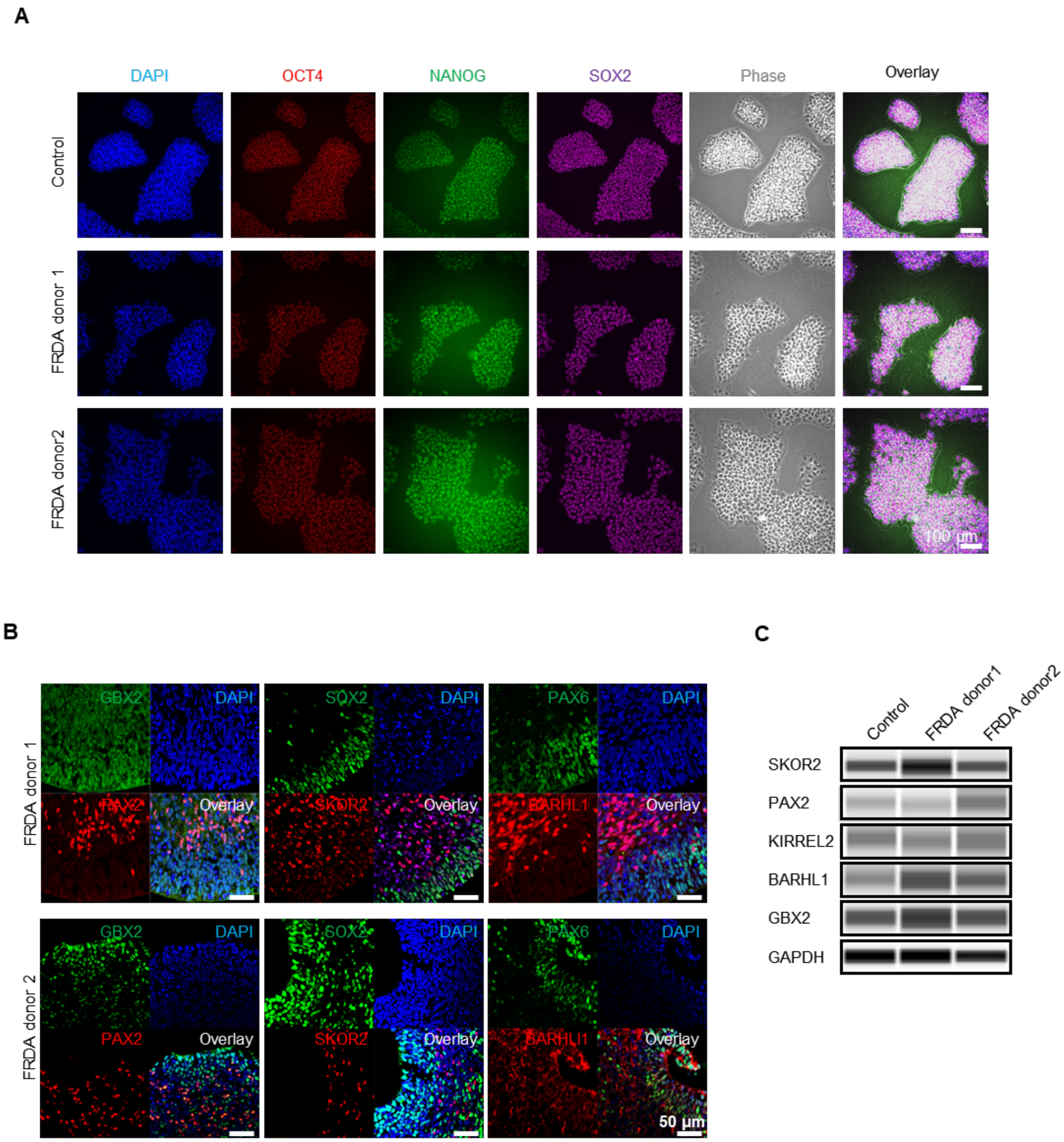

Figure S7. Characterization of iPSC lines and hCBOs from healthy individual (control) and FRDA patients

(A) Expression of pluripotency-associated markers OCT4, NANOG, and SOX2 in iPSCs from a healthy control and two FRDA donors.

(B) Immunohistochemical analysis showing normal hCBO generation and expression of typical cerebellar markers at day 35. No apparent differences were observed between control and FRDA donor iPSC lines.

(C) Western blot analysis showing primordial cerebellar markers (GBX2 for caudal region of mid-hindbrain boundary, BARHL1 for RL, and PAX2 for CPNE) expressed by gene-corrected, isogenic FRDA donor 2 iPSC clones at day 35 compared to day 0.

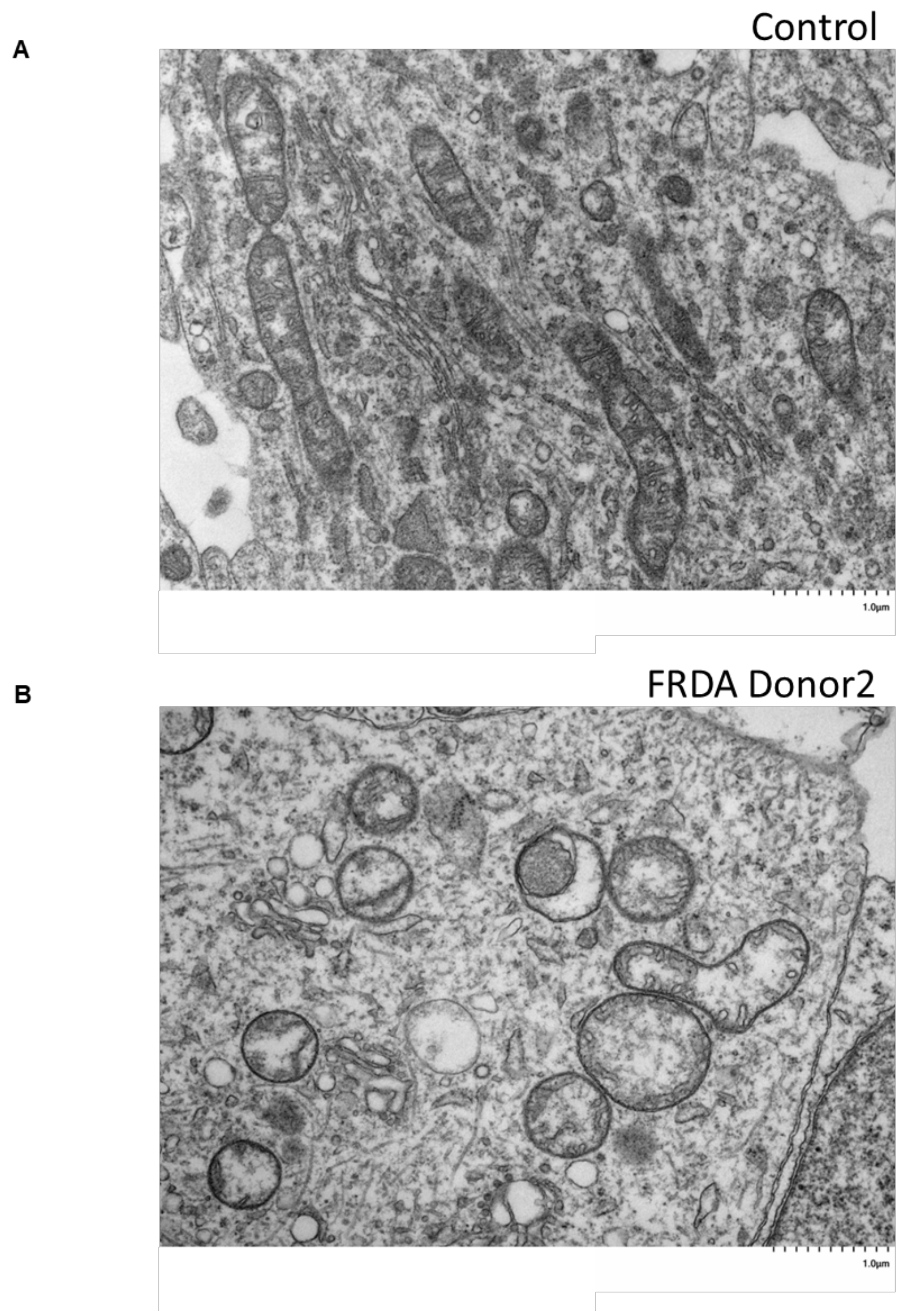

Figure S8. Ultrastructural analysis of mitochondria in normal and FRDA iPSC lines
(A, B) Higher magnification of electron microscopic images showing normal mitochondria (A) and mitochondria with pathological changes in FRDA (B). See also Figure 6J and 6K.

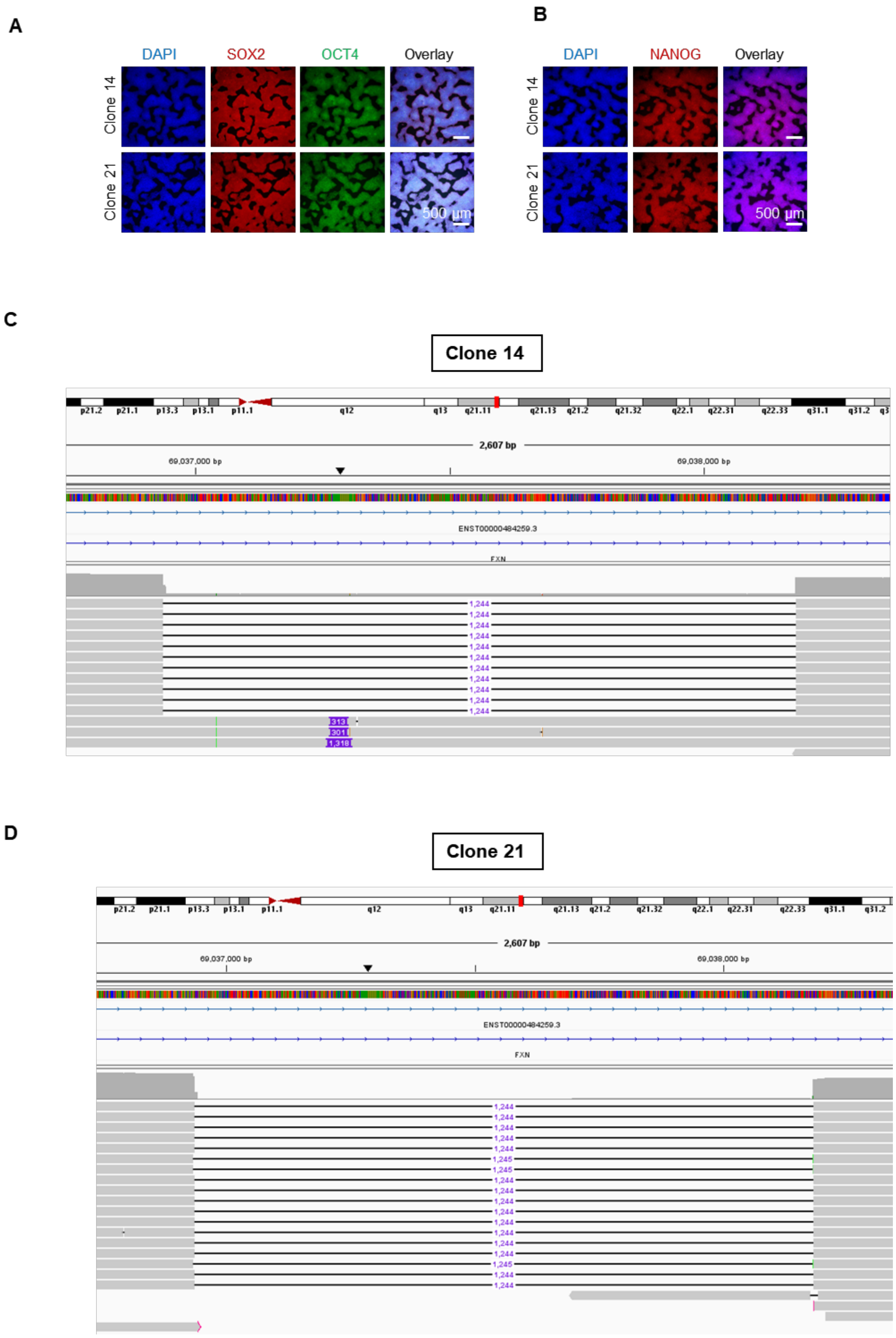

Figure S9. Characterization of isogenic gene-corrected FRDA iPSC lines

(A) Expression of pluripotency markers in two established cell clones of gene-corrected FRDA iPSC lines (FRDA donor 2).

(B-C) Visualization of FXN intron 1 targeted locus using the Integrated Genome Viewer (IGV) in the gene-corrected isogenic iPSCs from an FRDA patient (FRDA donor 2). PacBio long-read sequencing of the iPSC clones confirms excision in the FXN intron 1 locus (1244 bp + GAA repeats).

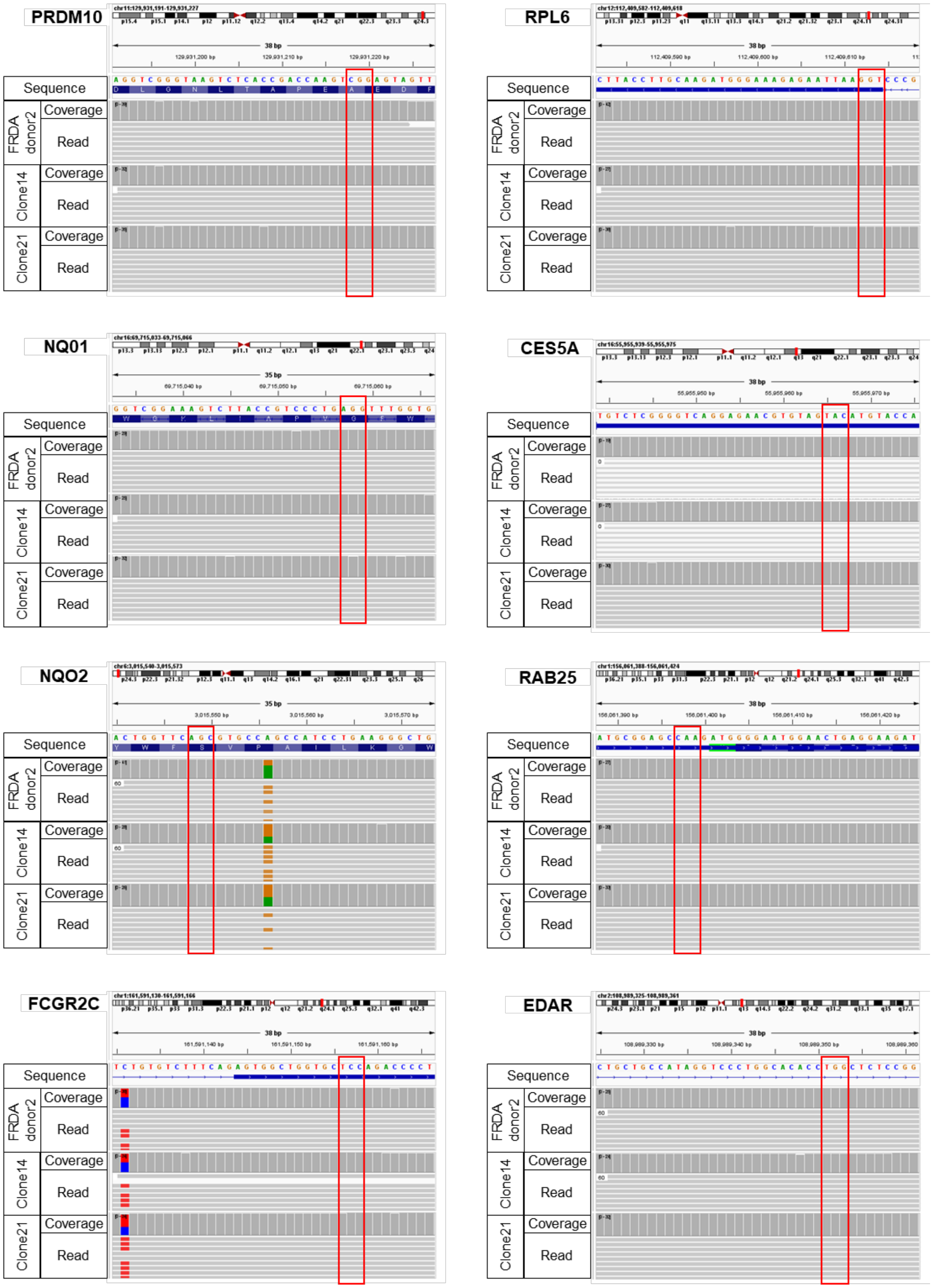

Figure S10. Analysis of the four highest ranked off-target genomic sites in the corrected, isogenic FRDA donor 2 iPSC clones

For 5' and 3' gRNAs utilized for the genomic deletion, the corrected isogenic FRDA donor 2 iPSC clones showed no off-target deletions in any of the predicted off-target sites. The PAM sequences are highlighted in the red box.

Clone 14

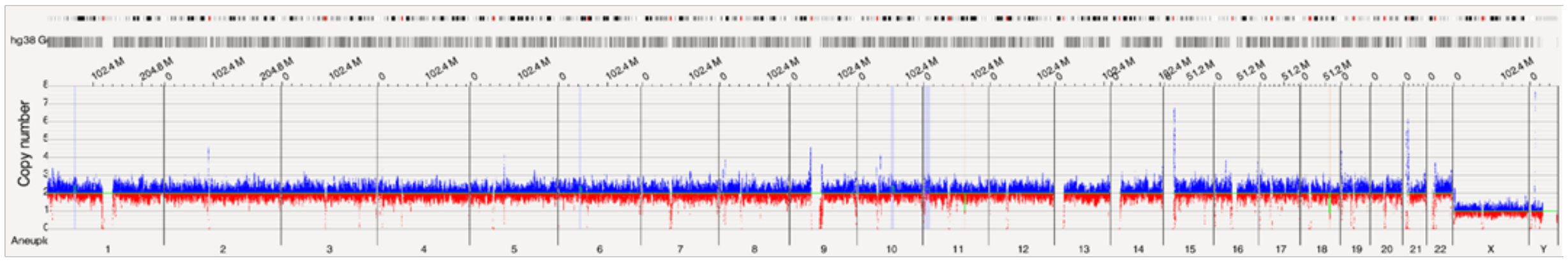

Clone 21

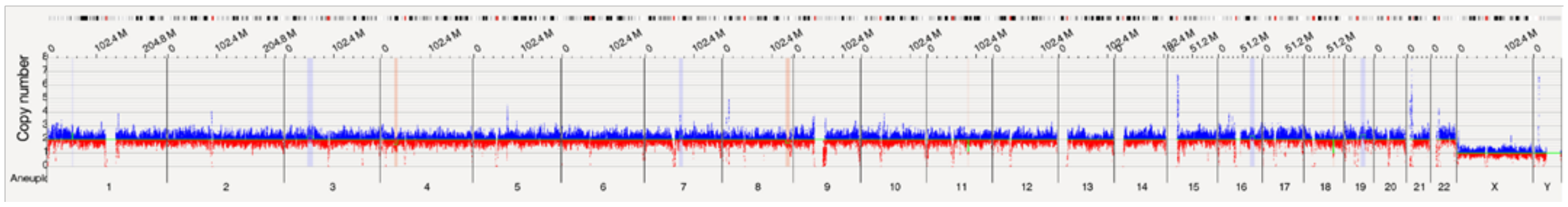

Figure S11. Copy number frequency plots of the gene-corrected, isogenic FRDA donor 2 iPSC clones identified by optical genome mapping

Plots showing typical copy number frequency across entire chromosomes, *i.e.*, two copy numbers in chromosomes 1-22 and 1 copy number in chromosomes X and Y, respectively.

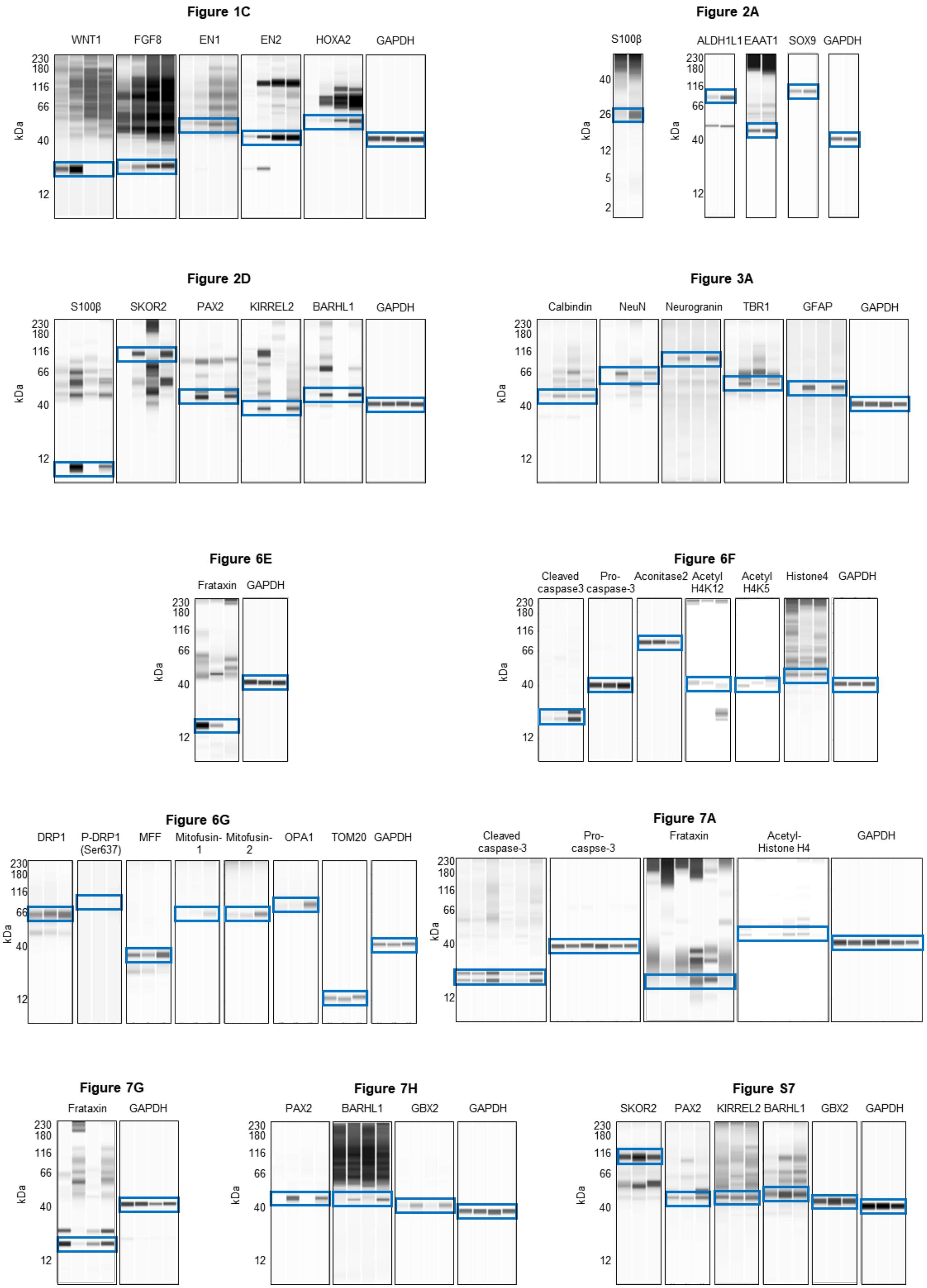

Figure S12. Uncropped and unedited western blot images

Figure 6D

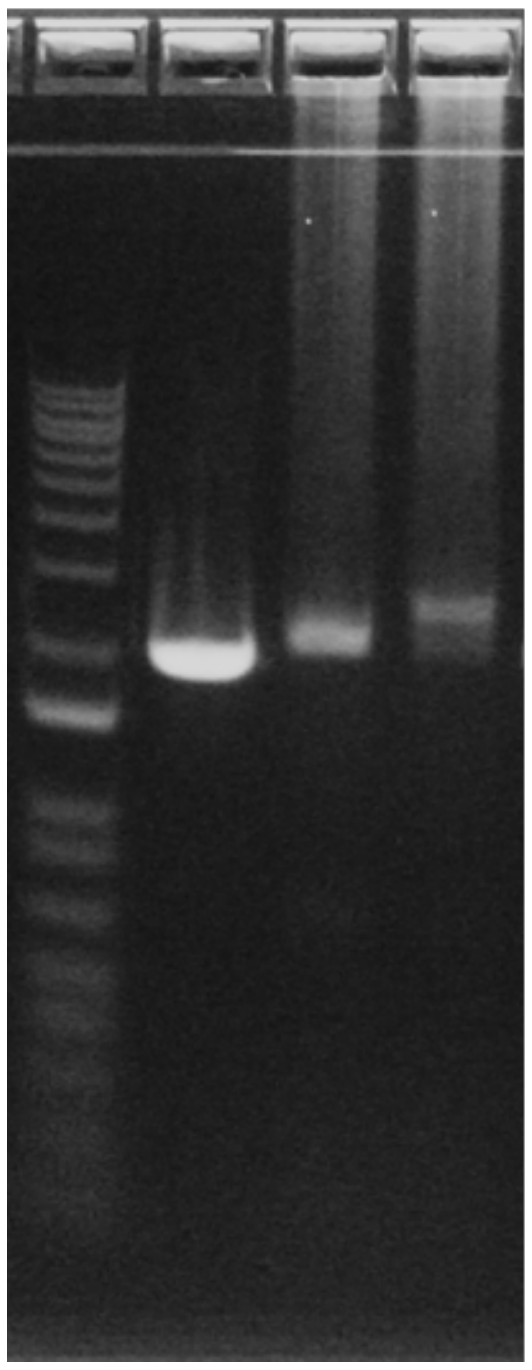

Figure 7D

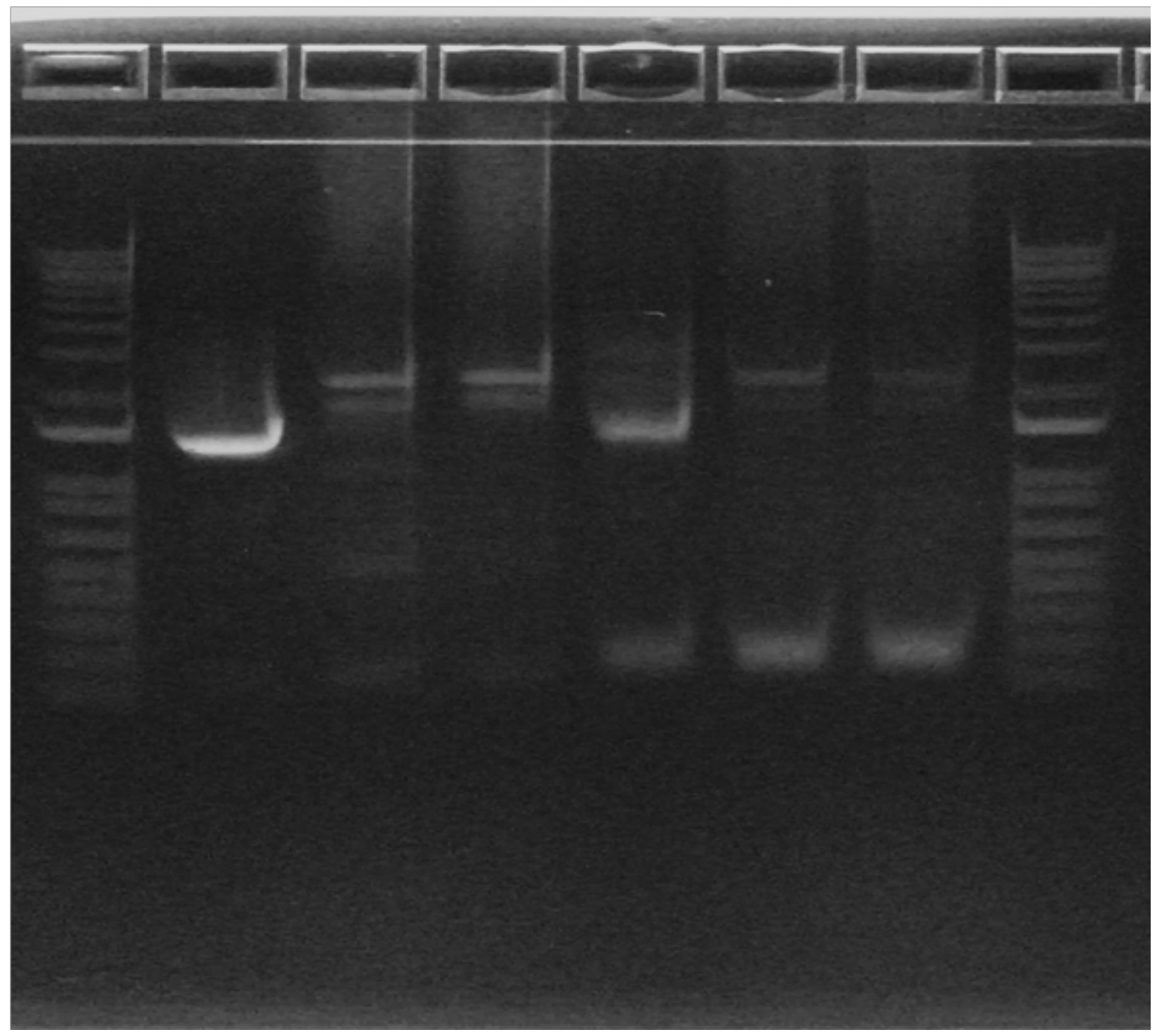

Figure S13. Uncropped and unedited gel electrophoresis images

Supplementary Table 1. Curated gene signatures for gene set scoring

**Region**	**Cell type / subclass**	**Marker gene**
Cerebellum	Bergmann glia	*ACSBG1*
Cerebellum	Bergmann glia	*ALDH1L1*
Cerebellum	Bergmann glia	*AQP4*
Cerebellum	Bergmann glia	*FABP7*
Cerebellum	Bergmann glia	*GLUL*
Cerebellum	Bergmann glia	*GPR37L1*
Cerebellum	Bergmann glia	*HOPX*
Cerebellum	Bergmann glia	*S100B*
Cerebellum	Bergmann glia	*SLC1A2*
Cerebellum	Bergmann glia	*SLC1A3*
Cerebellum	Bergmann glia	*SOX9*
Cerebellum	Deep cerebellar nuclei (excitatory/inhibitory)	*GAD1*
Cerebellum	Deep cerebellar nuclei (excitatory/inhibitory)	*GAD2*
Cerebellum	Deep cerebellar nuclei (excitatory/inhibitory)	*NEUROD1*
Cerebellum	Deep cerebellar nuclei (excitatory/inhibitory)	*NEUROD2*
Cerebellum	Deep cerebellar nuclei (excitatory/inhibitory)	*SLC17A6*
Cerebellum	Endothelial	*CLDN5*
Cerebellum	Endothelial	*KDR*
Cerebellum	Endothelial	*PECAM1*
Cerebellum	Golgi cell	*GAD1*
Cerebellum	Golgi cell	*GAD2*
Cerebellum	Golgi cell	*GRM2*
Cerebellum	Granule cell / lineage	*ATOH1*
Cerebellum	Granule cell / lineage	*CNTN2*
Cerebellum	Granule cell / lineage	*ETV1*
Cerebellum	Granule cell / lineage	*GABRA6*
Cerebellum	Granule cell / lineage	*KCNIP4*
Cerebellum	Granule cell / lineage	*NEUROD1*
Cerebellum	Granule cell / lineage	*PAX6*
Cerebellum	Granule cell / lineage	*SLC17A7*
Cerebellum	Granule cell / lineage	*TMEM266*
Cerebellum	Granule cell / lineage	*TUBB3*
Cerebellum	Granule cell / lineage	*ZIC1*
Cerebellum	Granule cell / lineage	*ZIC2*
Cerebellum	Microglia	*CX3CR1*
Cerebellum	Microglia	*P2RY12*
Cerebellum	Microglia	*TMEM119*
Cerebellum	Molecular layer interneuron (basket/stellate)	*GABRA1*
Cerebellum	Molecular layer interneuron (basket/stellate)	*GAD1*

Cerebellum	Molecular layer interneuron (basket/stellate)	*GAD2*
Cerebellum	Molecular layer interneuron (basket/stellate)	*KCNC1*
Cerebellum	Molecular layer interneuron (basket/stellate)	*NOS1*
Cerebellum	Molecular layer interneuron (basket/stellate)	*PAX2*
Cerebellum	Molecular layer interneuron (basket/stellate)	*PVALB*
Cerebellum	Molecular layer interneuron (basket/stellate)	*RELN*
Cerebellum	Oligodendrocyte / OPC	*MAG*
Cerebellum	Oligodendrocyte / OPC	*MBP*
Cerebellum	Oligodendrocyte / OPC	*MOG*
Cerebellum	Oligodendrocyte / OPC	*OLIG2*
Cerebellum	Oligodendrocyte / OPC	*PDGFRA*
Cerebellum	Oligodendrocyte / OPC	*PLP1*
Cerebellum	Oligodendrocyte / OPC	*SOX10*
Cerebellum	Pericyte / SMC	*ACTA2*
Cerebellum	Pericyte / SMC	*PDGFRB*
Cerebellum	Pericyte / SMC	*RGS5*
Cerebellum	Purkinje neuron	*ALDOC*
Cerebellum	Purkinje neuron	*CA8*
Cerebellum	Purkinje neuron	*CALB1*
Cerebellum	Purkinje neuron	*FOXP2*
Cerebellum	Purkinje neuron	*FOXP4*
Cerebellum	Purkinje neuron	*GRID2*
Cerebellum	Purkinje neuron	*ITPR1*
Cerebellum	Purkinje neuron	*LHX5*
Cerebellum	Purkinje neuron	*PCP2*
Cerebellum	Purkinje neuron	*PCP4*
Cerebellum	Purkinje neuron	*RORA*
Cerebellum	Purkinje neuron	*SKOR2*
Cerebellum	Purkinje neuron	*TRPC3*
Cerebellum	Unipolar brush cell (UBC)	*CALB2*
Cerebellum	Unipolar brush cell (UBC)	*EOMES*
Cerebellum	Unipolar brush cell (UBC)	*GRM1*
Cerebellum	Unipolar brush cell (UBC)	*PLCB4*
Forebrain	Choroid plexus epithelium	*AQP1*
Forebrain	Choroid plexus epithelium	*KRT18*
Forebrain	Choroid plexus epithelium	*TTR*
Forebrain	Cortex excitatory (layer-specific)	*BCL11B*
Forebrain	Cortex excitatory (layer-specific)	*CUX1*
Forebrain	Cortex excitatory (layer-specific)	*CUX2*
Forebrain	Cortex excitatory (layer-specific)	*POU3F2*
Forebrain	Cortex excitatory (layer-specific)	*RORB*

Forebrain	Cortex excitatory (layer-specific)	*SATB2*
Forebrain	Cortex excitatory (layer-specific)	*TBR1*
Forebrain	Cortex excitatory (layer-specific)	*TLE4*
Forebrain	Intermediate progenitor	*EOMES*
Forebrain	Interneuron (CGE-derived)	*PROX1*
Forebrain	Interneuron (CGE-derived)	*RELN*
Forebrain	Interneuron (CGE-derived)	*VIP*
Forebrain	Interneuron (MGE-derived)	*LHX6*
Forebrain	Interneuron (MGE-derived)	*NKX2-1*
Forebrain	Interneuron (MGE-derived)	*PVALB*
Forebrain	Interneuron (MGE-derived)	*SST*
Forebrain	Oligodendrocyte / OPC	*MAG*
Forebrain	Oligodendrocyte / OPC	*MBP*
Forebrain	Oligodendrocyte / OPC	*MOG*
Forebrain	Striatal TAC3 interneuron	*TAC3*
Forebrain	Striatal medium spiny neuron (MSN)	*BCL11B*
Forebrain	Striatal medium spiny neuron (MSN)	*DRD1*
Forebrain	Striatal medium spiny neuron (MSN)	*DRD2*
Forebrain	Striatal medium spiny neuron (MSN)	*FOXP1*
Forebrain	Striatal medium spiny neuron (MSN)	*GPR6*
Forebrain	Striatal medium spiny neuron (MSN)	*ISL1*
Forebrain	Striatal medium spiny neuron (MSN)	*MEIS2*
Forebrain	Striatal medium spiny neuron (MSN)	*PPP1R1B*
Forebrain	Telencephalon / cortical identity	*EMX1*
Forebrain	Telencephalon / cortical identity	*EMX2*
Forebrain	Telencephalon / cortical identity	*FOXG1*
Forebrain	Thalamus	*FOXP2*
Forebrain	Thalamus	*GBX2*
Forebrain	Thalamus	*SLC17A6*
Midbrain	Midbrain GABAergic neuron	*GAD1*
Midbrain	Midbrain GABAergic neuron	*GAD2*
Midbrain	Midbrain dopaminergic (mDA) neuron	*DDC*
Midbrain	Midbrain dopaminergic (mDA) neuron	*EN1*
Midbrain	Midbrain dopaminergic (mDA) neuron	*EN2*
Midbrain	Midbrain dopaminergic (mDA) neuron	*FOXA2*
Midbrain	Midbrain dopaminergic (mDA) neuron	*LMX1A*
Midbrain	Midbrain dopaminergic (mDA) neuron	*LMX1B*
Midbrain	Midbrain dopaminergic (mDA) neuron	*NR4A2*
Midbrain	Midbrain dopaminergic (mDA) neuron	*OTX2*
Midbrain	Midbrain dopaminergic (mDA) neuron	*PITX3*
Midbrain	Midbrain dopaminergic (mDA) neuron	*SLC18A2*

Midbrain	Midbrain dopaminergic (mDA) neuron	*SLC6A3*
Midbrain	Midbrain dopaminergic (mDA) neuron	*TH*
Midbrain	Midbrain glutamatergic neuron	*SLC17A6*
Midbrain	mDA A10 (VTA) subtype	*CALB1*
Midbrain	mDA A10 (VTA) subtype	*OTX2*
Midbrain	mDA A9 (SNc) subtype	*ALDH1A1*
Midbrain	mDA A9 (SNc) subtype	*KCNJ6*
Midbrain	mDA A9 (SNc) subtype	*SOX6*

Supplementary Table 2. List of primary and secondary antibodies.

Type	Antibody	Vendor	Catalog Number	Application	Dilution Factor
Primary	WNT1	Santa Cruz	sc-514531	Western blot	1:20
Primary	FGF8	Santa Cruz	sc-293479	Western blot	1:20
Primary	EN1	Santa Cruz	sc-398534	Western blot	1:20
Primary	EN2	Santa Cruz	sc29331	Western blot	1:20
Primary	Hoxa2	Novus Biologicals	nbp2-58865	Western blot	1:20
Primary	GAPDH	Santa Cruz	sc-3765062	Western blot	1:10000
Primary	SOX2	Abcam	ab79351	Immunostaining	1:100
Primary	SOX2	Millipore	ab5603	Immunostaining	1:100
Primary	TUJ1	Biolegend	801202	Immunostaining	1:200
Primary	BrdU	Thermo Fisher Scientific	835128	Immunostaining	1:50
Primary	Kirrel2	R&D Systems	mab2564	Immunostaining, western blot	1:100, 1:20
Primary	SKOR2	Atlas Antibodies	HPA046206	Immunostaining, western blot	1:100, 1:20
Primary	PAX2	Biolegend	901001	Immunostaining, western blot	1:100, 1:20
Primary	Barhl1	Novus Biologicals	NBP1-86513	Immunostaining, western blot	1:100, 1:20
Primary	S100β	Abcam	ab52642	Immunostaining, western blot	1:100,1:20
Primary	ATOH1	ABclonal	A11477	Immunostaining	1:100
Primary	ALDH1L1	Cell Signaling Technologies	CST85828	western blot	1:20
Primary	EAAT1	Cell Signaling Technologies	CST5684	western blot	1:20
Primary	SOX9	Abcam	ab185230	western blot	1:20
Primary	Calbindin	Abcam	ab75524	Immunostaining, western blot	1:500, 1:20
Primary	RORα	Biolegend	683102	Immunostaining	1:100
Primary	GRID2	Santa Cruz	SC-393437	Immunostaining	1:100
Primary	GABA	Sigma	A2052	Immunostaining	1:100
Primary	NeuN	Abcam	ab104224	Immunostaining	1:100
Primary	NeuN	Abcam	ab177487	Immunostaining, western blot	1:200, 1:50
Primary	SMI32	Biolegend	801701	Immunostaining	1:100
Primary	TBR1	Abcam	ab183032	Immunostaining, western blot	1:100, 1:20
Primary	Neurogranin	Millipore	ab5620	Immunostaining, western blot	1:200, 1:50
Primary	MAP2	Millipore	mab3418	Immunostaining	1:200
Primary	GFAP	Dako	z0334	Immunostaining, western blot	1:500, 1:20
Primary	OCT4	Santa Cruz	sc-5279	Immunostaining	1:200
Primary	Nanog	Cell Signaling Technologies	4903s	Immunostaining	1:200
Primary	SOX2	R&D Systems	AF2018	Immunostaining	1:100
Primary	GBX2	Abnova	H00002637	Immunostaining	1:50
Primary	PAX6	Biolegend	862002	Immunostaining	1:100
Primary	GBX2	R&D Systems	af4638	Western blot	1:20
Primary	Frataxin	Abcam	ab110328	Western blot	1:20
Primary	Cleaved caspase-3	Cell Signaling Technologies	9664s	Western blot	1:50
Primary	Pro-caspase-3	Cell Signaling Technologies	9665s	Western blot	1:50
Primary	aconitase 2	Abcam	ab110321	Western blot	1:20
Primary	Acetyl H4K12	Cell Signaling Technologies	CST13944	Western blot	1:20
Primary	Acetyl H4K5	Cell Signaling Technologies	CST8647	Western blot	1:20
Primary	Histone4	Cell Signaling Technologies	CST2935	Western blot	1:20
Primary	DRP1	Cell Signaling Technologies	8570	Western blot	1:20
Primary	P-DRP1 (Ser637)	Cell Signaling Technologies	6319	Western blot	1:20
Primary	MFF	Cell Signaling Technologies	84580	Western blot	1:20
Primary	Mitofusin-1	Cell Signaling Technologies	14739	Western blot	1:20
Primary	Mtofusin-2	Cell Signaling Technologies	11925	Western blot	1:20
Primary	OPA1	Cell Signaling Technologies	80471	Western blot	1:20
Primary	TOM20	Cell Signaling Technologies	42406	Western blot	1:20
Secondary	Donkey anti-mouse Alexa 488	Thermo Fisher Scientific	A-21202	Immunostaining	1:500
Secondary	Donkey anti-rabbit Alexa 568	Thermo Fisher Scientific	A-10042	Immunostaining	1:500
Secondary	Donkey anti-goat Alexa 647	Thermo Fisher Scientific	A-21447	Immunostaining	1:500
Secondary	Donkey anti-mouse Alexa 568	Thermo Fisher Scientific	A-10037	Immunostaining	1:500
Secondary	Donkey anti-rabbit Alexa 488	Thermo Fisher Scientific	A-21206	Immunostaining	1:500

Supplementary Table 3. List of primer sequences.

Target	Forward	Reverse
**FXN intron1 GAA expansion**	GAGGTCTAACCTCTAGCTGCTC	AAGCCCAATACGTGGCAG
**FXN CRISPR-Cas9 validation**	GGAGGGAACCGTCTGGGCAAAGG	CAATCCAG GACAGTCAGGGCTTT

Supplementary Table 4. Guide RNA sequences.

gRNA	Sequences
**gRNA1 (up)**	GG CGU ACC AGC CAC UCU GAA GUU UUA GAG CUA UGC U
**gRNA2 (down)**	CA AGA UGU GCA AGG GAA CUA GUU UUA GAG CUA UGC U

References

1 Atamian, A. *et al.* Human cerebellar organoids with functional Purkinje cells. *Cell Stem Cell* **31**, 39–51 (2024).

2 Amin, N. D. *et al.* Generating human neural diversity with a multiplexed morphogen screen in organoids. *Cell Stem Cell* **31**, 1831–1846 (2024).
