# Supplementary material for: Modeling Friedreich’s ataxia with Bergmann glia-enriched human cerebellar organoids
Source: Commun Biol. 2026 May 8;9:1235. doi: 10.1038/s42003-026-10180-5 (PMC13598134; doi:10.1038/s42003-026-10180-5)
Supplement: Supplementary file 2 [file 42003_2026_10180_MOESM2_ESM.pdf]

**Description of Additional Supplementary Files**

File name: Supplementary data 1
Description: Source data
