# Supplementary material for: Modeling Friedreich’s ataxia with Bergmann glia-enriched human cerebellar organoids
Source: Commun Biol. 2026 May 8;9:1235. doi: 10.1038/s42003-026-10180-5 (PMC13598134; doi:10.1038/s42003-026-10180-5)
Supplement: Supplementary file 3 [file 42003_2026_10180_MOESM4_ESM.pdf]

nature portfolio

Corresponding author(s): Ilyas Singec, Carlos Tristan, Seungmi Ryu

Last updated by author(s): 2025/06/30

Reporting Summary

Nature Portfolio wishes to improve the reproducibility of the work that we publish. This form provides structure for consistency and transparency in reporting. For further information on Nature Portfolio policies, see our Editorial Policies and the Editorial Policy Checklist.

Statistics

For all statistical analyses, confirm that the following items are present in the figure legend, table legend, main text, or Methods section.

n/a	Confirmed	
☐	☒	The exact sample size (*n*) for each experimental group/condition, given as a discrete number and unit of measurement
☐	☒	A statement on whether measurements were taken from distinct samples or whether the same sample was measured repeatedly
☐	☒	The statistical test(s) used AND whether they are one- or two-sided<br>*Only common tests should be described solely by name; describe more complex techniques in the Methods section.*
☒	☐	A description of all covariates tested
☒	☐	A description of any assumptions or corrections, such as tests of normality and adjustment for multiple comparisons
☐	☒	A full description of the statistical parameters including central tendency (e.g. means) or other basic estimates (e.g. regression coefficient) AND variation (e.g. standard deviation) or associated estimates of uncertainty (e.g. confidence intervals)
☐	☒	For null hypothesis testing, the test statistic (e.g. *F*, *t*, *r*) with confidence intervals, effect sizes, degrees of freedom and *P* value noted<br>*Give P values as exact values whenever suitable.*
☒	☐	For Bayesian analysis, information on the choice of priors and Markov chain Monte Carlo settings
☒	☐	For hierarchical and complex designs, identification of the appropriate level for tests and full reporting of outcomes
☐	☒	Estimates of effect sizes (e.g. Cohen's *d*, Pearson's *r*), indicating how they were calculated

Our web collection on statistics for biologists contains articles on many of the points above.

Software and code

Policy information about availability of computer code

Data collection	RNA Seq data were collected using the Illumina Novaseq 6000 platform. Whole genome sequencing data were collected using the PacBio Revio platform. Ultra-high molecular weight gDNA were mapped using Bionano Genomics Saphyr instrument.
Data analysis	Statistical tests were performed in GraphPad Prism 10.3.1 and R 3.6.0. Genome analysis was performed using Bionano Solve-v 3.7 and Access 1.7.2. A customized fully automated digital image analysis algorithm segmented each cell as an individual object and quantified the neuronal migration. SONY SH800 and Flow Jo 10.8.0 were used for ROS quantification.

For manuscripts utilizing custom algorithms or software that are central to the research but not yet described in published literature, software must be made available to editors and reviewers. We strongly encourage code deposition in a community repository (e.g. GitHub). See the Nature Portfolio guidelines for submitting code & software for further information.

Data

Policy information about availability of data

All manuscripts must include a data availability statement. This statement should provide the following information, where applicable:

- Accession codes, unique identifiers, or web links for publicly available datasets
- A description of any restrictions on data availability
- For clinical datasets or third party data, please ensure that the statement adheres to our policy

RNA-seq and whole genome sequencing data generated in this study can be found in the NCBI SRA under the Bioproject ID PRJNA1263825.

Research involving human participants, their data, or biological material

Policy information about studies with human participants or human data. See also policy information about sex, gender (identity/presentation), and sexual orientation and race, ethnicity and racism.

Reporting on sex and gender	n/a
Reporting on race, ethnicity, or other socially relevant groupings	n/a
Population characteristics	n/a
Recruitment	n/a
Ethics oversight	n/a

Note that full information on the approval of the study protocol must also be provided in the manuscript.

Field-specific reporting

Please select the one below that is the best fit for your research. If you are not sure, read the appropriate sections before making your selection.

☒ Life sciences ☐ Behavioural & social sciences ☐ Ecological, evolutionary & environmental sciences

For a reference copy of the document with all sections, see nature.com/documents/nr-reporting-summary-flat.pdf

Life sciences study design

All studies must disclose on these points even when the disclosure is negative.

Sample size	No statistical method was used to predetermine the sample size. Sample sizes for experiments were estimated based on the experience of the investigators and similar studies. Three or more independent replicates were performed for most experiments.
Data exclusions	No data are excluded.
Replication	The experimental findings were independently validated by multiple team members.
Randomization	Cells were imaged randomly to measure morphology and neuronal migration. All cells within a given field of view were analyzed, therefore the was no requirement for randomnization.
Blinding	Investigators were not blinded during collection or analysis because each assay is conducted by one investigator from the beginning to the end. Instead, the findings were confirmed by independent replications by multiple team members.

Reporting for specific materials, systems and methods

We require information from authors about some types of materials, experimental systems and methods used in many studies. Here, indicate whether each material, system or method listed is relevant to your study. If you are not sure if a list item applies to your research, read the appropriate section before selecting a response.

Materials & experimental systems

n/a	Involved in the study	
☐	☒	Antibodies
☐	☒	Eukaryotic cell lines
☒	☐	Palaeontology and archaeology
☒	☐	Animals and other organisms
☒	☐	Clinical data
☒	☐	Dual use research of concern
☒	☐	Plants

Methods

n/a	Involved in the study	
☒	☐	ChIP-seq
☐	☒	Flow cytometry
☒	☐	MRI-based neuroimaging

Antibodies

Antibodies used	WNT1 Santa Cruz sc-514531 Western blot 1:20<br>FGF8 Santa Cruz sc-293479 Western blot 1:20

EN1 Santa Cruz sc-398534 Western blot 1:20
EN2 Santa Cruz sc29331 Western blot 1:20
Hoxa2 Novus Biologicals nbp2-58865 Western blot 1:20
GAPDH Santa Cruz sc-3765062 Western blot 1:10000
SOX2 Abcam ab79351 Immunostaining 1:100
SOX2 Millipore ab5603 Immunostaining 1:100
TUJ1 Biolegend 801202 Immunostaining 1:200
Kirrel2 R&D Systems mab2564 Immunostaining, western blot 1:100, 1:20
SKOR2 Atlas Antibodies HPA046206 Immunostaining, western blot 1:100, 1:20
PAX2 Biolegend 901001 Immunostaining, western blot 1:100, 1:20
Barhl1 Novus Biologicals NBP1-86513 Immunostaining, western blot 1:100, 1:20
S100β Abcam ab52642 Immunostaining, western blot 1:100,1:20
ATOH1 ABclonal A11477 Immunostaining 1:100
ALDH1L1 Cell Signaling Technologies CST85828 western blot 1:20
EAAT1 Cell Signaling Technologies CST5684 western blot 1:20
SOX9 Abcam ab185230 western blot 1:20
Calbindin Abcam ab75524 Immunostaining, western blot 1:500, 1:20
GABA Sigma A2052 Immunostaining 1:100
NeuN Abcam ab104224 Immunostaining 1:100
NeuN Abcam ab177487 Immunostaining, western blot 1:200, 1:50
SMI32 Biolegend 801701 Immunostaining 1:100
TBR1 Abcam ab183032 Immunostaining, western blot 1:100, 1:20
Neurogranin Millipore ab5620 Immunostaining, western blot 1:200, 1:50
MAP2 Millipore mab3418 Immunostaining 1:200
GFAP Dako z0334 Immunostaining, western blot 1:500, 1:20
OCT4 Santa Cruz sc-5279 Immunostaining 1:200
Nanog Cell Signaling Technologies 4903s Immunostaining 1:200
SOX2 R&D Systems AF2018 Immunostaining 1:100
GBX2 Abnova H00002637 Immunostaining 1:50
PAX6 Biolegend 862002 Immunostaining 1:100
GBX2 R&D Systems af4638 Western blot 1:20
Frataxin Abcam ab110328 Western blot 1:20
Cleaved caspase-3 Cell Signaling Technologies 9664s Western blot 1:50
Pro-caspase-3 Cell Signaling Technologies 9665s Western blot 1:50
aconitase 2 Abcam ab110321 Western blot 1:20
Acetyl H4K12 Cell Signaling Technologies CST13944 Western blot 1:20
Acetyl H4K5 Cell Signaling Technologies CST8647 Western blot 1:20
Histone4 Cell Signaling Technologies CST2935 Western blot 1:20
DRP1 Cell Signaling Technologies 8570 Western blot 1:20
P-DRP1 (Ser637) Cell Signaling Technologies 6319 Western blot 1:20
MFF Cell Signaling Technologies 84580 Western blot 1:20
Mitofusin-1 Cell Signaling Technologies 14739 Western blot 1:20
Mtofusin-2 Cell Signaling Technologies 11925 Western blot 1:20
OPA1 Cell Signaling Technologies 80471 Western blot 1:20
TOM20 Cell Signaling Technologies 42406 Western blot 1:20
Donkey anti-mouse Alexa 488 Thermo Fisher Scientific A-21202 Immunostaining 1:500
Donkey anti-rabbit Alexa 568 Thermo Fisher Scientific A-10042 Immunostaining 1:500
Donkey anti-goat Alexa 647 Thermo Fisher Scientific A-21447 Immunostaining 1:500
Donkey anti-mouse Alexa 568 Thermo Fisher Scientific A-10037 Immunostaining 1:500
Donkey anti-rabbit Alexa 488 Thermo Fisher Scientific A-21206 Immunostaining 1:500

Validation

WNT1 Santa Cruz sc-514531 https://www.scbt.com/p/wnt-1-antibody-e-10
FGF8 Santa Cruz sc-293479 https://www.scbt.com/p/fgf-8-antibody-2a10
EN1 Santa Cruz sc-398534 https://www.scbt.com/p/en1-antibody-c19
EN2 Santa Cruz sc29331 https://www.scbt.com/p/en2-antibody-a-3
Hoxa2 Novus Biologicals nbp2-58865 https://www.novusbio.com/products/hoxa2-antibody_nbp2-58865
GAPDH Santa Cruz sc-3765062 https://www.scbt.com/p/gapdh-antibody-mab374
SOX2 Abcam ab79351 https://www.abcam.com/sox2-antibody-9-9-3-ab79351.html
SOX2 Millipore ab5603 https://www.emdmillipore.com/US/en/product/ab5603
TUJ1 Biolegend 801202 https://www.biolegend.com/en-us/products/beta-iii-tubulin-neuronal-tuj1-antibody-801202
Kirrel2 R&D Systems mab2564 https://www.rndsystems.com/products/kirrel2-antibody_mab2564
SKOR2 Atlas Antibodies HPA046206 https://www.proteinatlas.org/ENSG00000154249-SKOR2/antibody
PAX2 Biolegend 901001 https://www.biolegend.com/en-us/products/pax2-antibody-901001
Barhl1 Novus Biologicals NBP1-86513 https://www.novusbio.com/products/barhl1-antibody_nbp1-86513
S100β Abcam ab52642 https://www.abcam.com/s100b-antibody-ab52642.html
ATOH1 ABclonal A11477 https://en.abclonal.com/AToh1-antibody-A11477.html
ALDH1L1 Cell Signaling Technologies CST85828 https://www.cellsignal.com/products/antibodies/aldh1l1-antibody/85828
EAAT1 Cell Signaling Technologies CST5684 https://www.cellsignal.com/products/antibodies/eaac1-glutamate-transporter-antibody/5684
SOX9 Abcam ab185230 https://www.abcam.com/sox9-antibody-ab185230.html
Calbindin Abcam ab75524 https://www.abcam.com/calbindin-antibody-ab75524.html
GABA Sigma A2052 https://www.sigmaaldrich.com/catalog/product/sigma/a2052
NeuN Abcam ab104224 https://www.abcam.com/neun-antibody-ab104224.html
NeuN Abcam ab177487 https://www.abcam.com/neun-antibody-ab177487.html
SMI32 Biolegend 801701 https://www.biolegend.com/en-us/products/neurofilament-heavy-chain-antibody-smi-32-801701
TBR1 Abcam ab183032 https://www.abcam.com/tbr1-antibody-ab183032.html

Neurogranin Millipore ab5620 https://www.emdmillipore.com/US/en/product/neurogranin-antibody-ab5620
MAP2 Millipore mab3418 https://www.emdmillipore.com/US/en/product/map2-antibody-mab3418
GFAP Dako z0334 https://www.agilent.com/en/product/immunohistochemistry-reagents/gfap-antibody-z0334–epitope-06-347
OCT4 Santa Cruz sc-5279 https://www.scbt.com/p/oct4-antibody-h134
Nanog Cell Signaling Technologies 4903s https://www.cellsignal.com/products/antibodies/nanog-antibody/4903
SOX2 R&D Systems AF2018 https://www.rndsystems.com/products/sox2-antibody_af2018
GBX2 Abnova H00002637 https://www.abnova.com/gbx2-antibody-n-pachyderma_h00002637-m01
PAX6 Biolegend 862002 https://www.biolegend.com/en-us/products/pax6-antibody-862002
GBX2 R&D Systems af4638 https://www.rndsystems.com/products/gbx2-antibody_af4638
Frataxin Abcam ab110328 https://www.abcam.com/frataxin-antibody-ab110328.html
Cleaved caspase-3 Cell Signaling Technologies 9664s https://www.cellsignal.com/products/antibodies/cleaved-caspase-3-antibody/9664
Pro-caspase-3 Cell Signaling Technologies 9665s https://www.cellsignal.com/products/antibodies/pro-caspase-3-antibody/9665
aconitase 2 Abcam ab110321 https://www.abcam.com/aconitase-2-antibody-ab110321.html
Acetyl H4K12 Cell Signaling Technologies CST13944 https://www.cellsignal.com/products/antibodies/acetyl-histone-h4k12-antibody/13944
Acetyl H4K5 Cell Signaling Technologies CST8647 https://www.cellsignal.com/products/antibodies/acetyl-histone-h4k5-antibody/8647
Histone4 Cell Signaling Technologies CST2935 https://www.cellsignal.com/products/antibodies/histone-h4-antibody/2935
DRP1 Cell Signaling Technologies 8570 https://www.cellsignal.com/products/antibodies/drp1-antibody/8570
P-DRP1 (Ser637) Cell Signaling Technologies 6319 https://www.cellsignal.com/products/antibodies/phospho-drp1-ser637-antibody/6319
MFF Cell Signaling Technologies 84580 https://www.cellsignal.com/products/antibodies/mff-antibody/84580
Mitofusin-1 Cell Signaling Technologies 14739 https://www.cellsignal.com/products/antibodies/mitofusin-1-antibody/14739
Mtofusin-2 Cell Signaling Technologies 11925 https://www.cellsignal.com/products/antibodies/mitofusin-2-antibody/11925
OPA1 Cell Signaling Technologies 80471 https://www.cellsignal.com/products/antibodies/opa1-antibody/80471
TOM20 Cell Signaling Technologies 42406 https://www.cellsignal.com/products/antibodies/tom20-antibody/42406
Donkey anti-mouse Alexa 488 Thermo Fisher Scientific A-21202 https://www.thermofisher.com/antibody/A-21202
Donkey anti-rabbit Alexa 568 Thermo Fisher Scientific A-10042 https://www.thermofisher.com/antibody/A-10042
Donkey anti-goat Alexa 647 Thermo Fisher Scientific A-21447 https://www.thermofisher.com/antibody/A-21447
Donkey anti-mouse Alexa 568 Thermo Fisher Scientific A-10037 https://www.thermofisher.com/antibody/A-10037
Donkey anti-rabbit Alexa 488 Thermo Fisher Scientific A-21206 https://www.thermofisher.com/antibody/A-21206

Eukaryotic cell lines

Policy information about cell lines and Sex and Gender in Research

Cell line source(s)	Human induced pluripotent stem cells (iPSCs) were obtained from NIGMS Human Genetic Cell Repository at the Coriell Institute for Medical Research (GM25256, GM23404, GM23903) and NIH Common Fund (NCRM5). Human embryonic stem cells (hESCs) were obtained from WiCell Research Institute (WA09).
Authentication	Cells were not further authenticated in our lab.
Mycoplasma contamination	All cell lines were tested to be mycoplasma negative.
Commonly misidentified lines (See ICLAC register)	No commonly misidentified cell lines were used.

Plants

Seed stocks	n/a
Novel plant genotypes	n/a
Authentication	n/a

Flow Cytometry

Plots

Confirm that:

☒ The axis labels state the marker and fluorochrome used (e.g. CD4-FITC).

☒ The axis scales are clearly visible. Include numbers along axes only for bottom left plot of group (a 'group' is an analysis of identical markers).

☒ All plots are contour plots with outliers or pseudocolor plots.

☒ A numerical value for number of cells or percentage (with statistics) is provided.

Methodology

Sample preparation	For ROS quantification, day 60 hCBOs were washed twice in DPBS (Thermo Fisher Scientific) and dissociated into a single-cell suspension using Embryoid Body Dissociation Kit (Miltenyi Biotech) and gentleMACS Dissociator (Miltenyi Biotech) in a gentleMACS C Tube (Miltenyi Biotech) following the manufacturer's protocol. The cell suspension was filtered through a 70-µm strainer (Miltenyi Biotech) to remove cell debris and clumps. The strained cell suspension was centrifuged at 300 g for 5 min and stained with H2DCFDA reagent (Thermo Fisher Scientific) according to the manufacturer's protocol.
Instrument	SONY SH800
Software	FlowJo 10.8.0
Cell population abundance	*Describe the abundance of the relevant cell populations within post-sort fractions, providing details on the purity of the samples and how it was determined.*
Gating strategy	To measure the mean fluorescence intensity of the total cell population, the cells were not gated.

☐ Tick this box to confirm that a figure exemplifying the gating strategy is provided in the Supplementary Information.
